# Rate of entropy model for irreversible processes in living systems

**DOI:** 10.1038/s41598-017-09530-5

**Published:** 2017-08-22

**Authors:** R. Zivieri, N. Pacini, G. Finocchio, M. Carpentieri

**Affiliations:** 1 Department of Physics and Earth Sciences and Consorzio Nazionale Interuniversitario per le Scienze Fisiche della Materia, Unit of Ferrara, University of Ferrara, via G. Saragat 1, Ferrara, I-44122 Italy; 2 Department of Mathematical and Computer Sciences, Physical Sciences and Earth Sciences, University of Messina, V.le F. D’Alcontres, 31, 98166 Messina, Italy; 3 Department of General Surgery and Senology, University Hospital Company, Policlinico Vittorio Emanuele, via S. Citelli 6, 95124 Catania, Italy; 4 Department of Electrical and Information Engineering, Politecnico di Bari, via E. Orabona 4, Bari, I-70125 Italy

## Abstract

In living systems, it is crucial to study the exchange of entropy that plays a fundamental role in the understanding of irreversible chemical reactions. However, there are not yet works able to describe in a systematic way the rate of entropy production associated to irreversible processes. Hence, here we develop a theoretical model to compute the rate of entropy in the minimum living system. In particular, we apply the model to the most interesting and relevant case of metabolic network, the glucose catabolism in normal and cancer cells. We show, (i) the rate of internal entropy is mainly due to irreversible chemical reactions, and (ii) the rate of external entropy is mostly correlated to the heat flow towards the intercellular environment. The future applications of our model could be of fundamental importance for a more complete understanding of self-renewal and physiopatologic processes and could potentially be a support for cancer detection.

## Introduction

The irreversible processes in living systems are fundamental for determining the autopoietic life development and lead to entropy production in living systems^1–3^. Examples of irreversible chemical reactions that represent basic processes both for prebiotic life and for life development and maintenance are sodium/potassium pump, *β*-oxidation of fatty acids, protein catabolic process, glucose catabolism, etc. The full understanding of the non-equilibrium thermodynamics of irreversible reactions is important for the origin and the maintenance of life^4, 5^. Very interestingly, while mechanical phenomena are invariant under time-reversal symmetry, thermodynamic ones introduce an arrow of time breaking this symmetry. This behaviour is naturally linked to the concept of entropy^6^ that, according to its first definition by Clausius and Boltzmann^7, 8^, describes the thermodynamics of catabolic processes.

Recent works have shown that there is a link between the irreversible processes in the metabolic network, such as glucose catabolism, and epigenetic and gene network^9–13^, and that the entropy definition introduced by Clausius and Boltzmann^7, 8^ is equivalent to Shannon information entropy^14–19^. Since the 20 s^20, 21^, it is known that almost all cancer cells show a deep alteration of their metabolic networks with a marked shift towards the lactic acid fermentation or aerobic glycolysis at the expense of oxidative phosphorylation (OXPHO). Interestingly, the relation between the glucose catabolism and the gene and epigenetic network strengthens during the cancer development^22–28^, likewise there has been a wide use of the intensity of aerobic glycolysis in diagnostics with correlations to cancer prognosis^29–31^.

Although it is well-known the crucial role played by irreversible reactions in living systems, a self-consistent description of the calculation of the entropy exchanges between the cell and the environment is missing. This is because the quantitative description of cell thermodynamics is still incomplete. Furthermore, the important quantitative aspects related to the time behaviour of entropy have not been taken into account. Indeed, it has emphasized the exergy concept regardless of the ATP yield and of coupling between exo- and endo-ergonic reactions^32, 33^. In other words, the concept of rate of entropy is different from the concepts of finite variations of entropy and Gibbs free energy between reactants and products. Moreover, some *in vitro* experiments and biophysical theoretical investigations have proved that the heat flow is not only the simple effect of degradation of energy but plays also an important role in the ionic transport and in the interactions among biomolecules such as DNA, proteins and so on^34–36^.

Here, we formulate a model relying on the Clausius definition of entropy and on Prigogine’s approach able to give a full thermodynamic description of the rate of entropy density production associated to irreversible chemical processes in living systems. This model considers: i) the time dependence of the entropy exchanges in the cell and out of it, and ii) the effect of the cell size on the rate of entropy density production. We apply the model to the glucose catabolism in a typical human tissue represented by breast cells. The choice of glucose catabolism is essentially twofold. First, several recent works have shown its important role for the different pathways driving cancer development^37–39^. Second, the glucose catabolism is, among the irreversible reactions occurring in cells, the one having the highest frequency of occurrence and the largest entropy production^40^. The key result of our work on glucose catabolism is that the diffusion of chemical species gives the largest contribution to the rate of internal entropy density both in normal and cancer cells, while the rate of external entropy density, mainly due to heat diffusion, has an enhancement in cancer cells. Although most of cancer therapy is mainly pharmacological, a wider knowledge of the physical processes like, for example, the observation of temperature differences between the intracellular and the intercellular environment is important for developing new prospects in cancer detection^41–43^. This latter aspect is widely coherent with the experimental trials and clinical data, and could open new perspectives on the hyperthermia cancer therapy^44, 45^.

### Model for the calculation of the rate of entropy in living systems

We have studied the cell as an open thermodynamic system in local equilibrium. Hence, starting from Prigogine’s approach^46^ combined with diffusion equations valid for heat and mass transport, we have computed the rate of entropy density production $r=ds/dt$ for a cell with *s* entropy density and *t* time (see Methods). The exchange of entropy occurs at two different levels, in the cell interior and with the intercellular environment, hence $r={r}_{i}+{r}_{e}$ where ${r}_{i}=d{s}_{i}/dt$ is the rate of internal entropy density production (RIEDP) where the subscript “*i*” denotes internal and ${r}_{e}=d{s}_{e}/dt$ is the rate of external entropy density production (REEDP) where the subscript “*e*” denotes external. To calculate *r*_*i*_ we have made the following main assumptions:

(i) Cells are assumed of cubic shape having volume *V*_cell_ = *L* ^3^ with *L* the side of the average cube;
(ii) Flows are along a preferential direction (1D approximation) (*x* direction here);
(iii) Irreversible processes start in the centre of the cytoplasm region, namely for *x* = *L*/2;
(iv) Cross-effects in determining either the heat flow or the mass flow are absent;
(v) The volume of the cell nucleus is negligible.

The model is applicable to any kind of chemical reaction involved in the metabolic cellular activity of living systems.

Assumption (i) allows the study of cells of various shapes (e.g. spherical, cylindrical, elliptical and so on) represented as cubes having volumes equivalent to those of the specific shape studied. In particular, assumption (i) applies to several epithelium tissues like, for example, the breast epithelium and the exocrine glands epithelium whose shape is columnar^47^. As cellular processes occur prevalently along a preferential direction^48^, typically assumption (ii) is valid in real systems. Assumption (iii) is supported by the fact that mytochondria, where most of the catabolic processes in normal and cancer cells occur (e.g. Krebs cycle, *β*-ossidation and part of protein catabolism), are approximately placed in the perinuclear region^49^. Moreover, in cancer cells also lactic acid fermentation takes place in the mytocondrial region. However, note that in some cases a few catabolic reactions like, for instance chemotaxis, occur in cell peripheral zones. In these specific cases, it would be enough to make a translation of the origin of *L*/2 to the cell border. Assumption (iv) is consistent in the presence of polarization effects occurring in cells^48, 50^. Assumption (v) is reasonable in most of the cells being the volume of the nucleus much smaller than *V*_cell_^49^. By taking into account all the above assumptions, we have computed ${r}_{i}(x,t)={r}_{iQ}(x,t)+{r}_{iD}(x,t)+{r}_{ir}(x,t)$ where *r*_*i Q*_ (*x*,*t*) is due to heat flow with the subscript “*Q*” labelling heat, *r*_*i D*_ (*x*,*t*) is caused by molecules diffusion with the subscript “*D*” standing for diffusion and *r*_*i r*_ (*x*,*t*), with the subscript“*r*” indicating reactions, is directly related to irreversible chemical reactions. All terms are products between thermodynamic forces and flows generated by them. In explicit form, for any irreversible reactions, the contribution due to heat flow *r*_*i Q*_ (*x*,*t*) is given by

1 $${r}_{iQ}\,(x,t)=\alpha \,\frac{{[\sum _{n=1}^{\infty }(\cos [(2n-1)\frac{\pi }{L}x]{e}^{-\kappa {(2n-1)}^{2}\frac{{\pi }^{2}}{{L}^{2}}t})]}^{2}{e}^{-\frac{t}{\tau }}}{{[T(x,t)]}^{2}}$$

Here, $\alpha =16pK\frac{{T}_{0}^{2}}{{L}^{2}}$ with *p* the weight associated to the specific chemical reaction evaluated as a function of the occurrence frequency of the reaction, *K* is the thermal conductivity, *κ* is the thermal diffusivity and *τ* is a typical cell decay time. The term $T(x,t)=\frac{4{T}_{0}}{{\pi }}\sum _{n=1}^{\infty }$ $(\sin [(2n-1)\frac{\pi }{L}x]/(2n-1)\,{e}^{-\kappa {(2n-1)}^{2}\frac{{\pi }^{2}}{{L}^{2}}t})$ is the temperature distribution (see Supplementary information, Section 2, equation (S3) and equation (S5)) showing that the temperature inside the cell has a weak spatial dependence coherently with the data obtained by means of fluorescent technique^43^. The cosine series at the numerator results both from the calculation of the thermodynamic force calculated as the gradient of the inverse of the temperature distribution and the heat flow proportional to the spatial derivative of *T* (*x*,*t*) (see Supplementary information, Section 2, equation (S6) and equation (S7), respectively).

Instead, for every irreversible process, *r*_*i D*_ (*x*,*t*) takes the form

2 $${{r}}_{{iD}}\,(x{,}t){\boldsymbol{=}}\beta \,\frac{(x-L/2){e}^{-\frac{t}{\tau }}}{{t}^{3/2}}(\sum _{k=1}^{N}({u}_{k}\frac{{N}_{mk}}{4\sqrt{{D}_{k}}}{e}^{-\frac{{(x-L/2)}^{2}}{4{D}_{k}t}}))(\frac{(\sum _{n=1}^{\infty }(\cos \,[(2n-1)\frac{\pi }{L}x]{e}^{-\kappa {(2n-1)}^{2}\frac{{\pi }^{2}}{{L}^{2}}t}){e}^{-|x-L/2|/L})}{{[T(x,t)]}^{2}})$$

Here, $\beta =\frac{1}{{\pi }^{\frac{1}{2}}}\frac{1}{{V}_{{\rm{cell}}}}{T}_{0}\,$, *N* is the number of chemical species involved in the reaction and ${\mu }_{k}(x,t)={u}_{k}{e}^{-(|x-L/2|/L+t/\tau )}$ is the *k* ^th^ chemical potential with ${u}_{k}$ the partial molar energy, *D*_*k*_ is the corresponding mass diffusion coefficient and *N*_*m k*_ is the number of moles of the *k*_th_ chemical species. It is ${e}^{-(x-L/2)/L}$ (${e}^{-(L/2-x)/L}$) for 0 ≤ *x* ≤ *L*/2 (*L*/2 ≤ *x* ≤ *L*). The cosine series at the numerator results from the thermodynamic force evaluated as the gradient of the ratio between the chemical potential and the temperature distribution, while the Gaussian distribution comes from the diffusion flow proportional to the derivative of the number of moles per unit volume (see Supplementary information, Section 2, equation (S11) and equation (S15), respectively).

Finally, *r*_*i r*_ (*x*,*t*) is

3 $${r}_{ir}(x,t)=-\gamma \frac{1}{{V}_{{\rm{c}}{\rm{e}}{\rm{l}}{\rm{l}}}^{p+q}}\frac{{k}_{{\rm{k}}{\rm{i}}{\rm{n}}}(\sum _{k=1}^{N}{\nu }_{k}{u}_{k}{e}^{-(|x-L/2|/L+t/\tau )}){N}_{m{\rm{A}}\,{\rm{r}}{\rm{e}}{\rm{a}}{\rm{g}}}^{p}{N}_{m{\rm{B}}\,{\rm{r}}{\rm{e}}{\rm{a}}{\rm{g}}}^{q}}{{[T(x,t)]}^{2}}$$

where,$\,\gamma =\frac{4}{\pi }{T}_{0}$, ${k}_{{\rm{kin}}}$ is the pathway kinetic constant of the given irreversible process with “kin” standing for kinetic, *ν*_*k*_ are stoichiometric coefficients, ${\mu }_{k}(x,t)={u}_{k}{e}^{-(|x-L/2|/L+t/\tau )}$ is the chemical potential of the *k* ^th^ species. Here *p* = 0, 1, 2, *q* = 0, 1, 2 and *p* + *q* = 1, 2 for first- and second-order irreversible chemical reactions, respectively and $\frac{{N}_{m{\rm{A}}{\rm{reag}}}}{{V}_{{\rm{cell}}}}$ ($\frac{{N}_{m{\rm{B}}{\rm{reag}}}}{{V}_{{\rm{cell}}}}$) is the molar concentration of reagent A (B) taking the volume of the solution equal to *V*_cell_. The dependence on the chemical potential and on the molar concentrations at the numerator results from the definition of affinity and of the velocity of the given reaction (see Supplementary information, Section 2, equation (S20), equation (S21) and equation (S22), respectively). For the detailed derivation of equations (1–3), see Supplementary Information, Section 2.

We now discuss $\,{r}_{e}(x,t)$ calculated by taking into account assumptions (i), (ii) and (v). We express ${r}_{e}(x,t)={r}_{eQ}(x,t)+{r}_{e{\rm{exch}}}\,(x,t)$, where *r*_*e Q*_ (*x*,*t*) is the contribution due to heat released by the cell in the intercellular environment, while *r*_*e* exch_ (*x*,*t*), with “exch” standing for exchanges, is the one related to exchanges of matter with the intercellular environment.

The derivation of *r*_*e Q*_ (*x*,*t*) lies on simple thermodynamic considerations. Specifically, we have used the first principle of thermodynamics for the expression of the heat released, the solution of heat equation in the intercellular environment (see Supplementary information, Section 3 equation (S29)) and exploiting the analogy of the behaviour of the cellular system mainly composed by water with that of an ideal gas. As in the case of a monoatomic gas, we derive the cell internal energy starting from its partition function that has a direct thermodynamic relation with the internal energy. It turns out that *r*_*e Q*_ (*x*,*t*) reads

4 $${r}_{eQ}(x,t)\simeq \delta \frac{{N}_{m{\rm{p}}{\rm{r}}}}{\kappa }\frac{{(x-L)}^{2}}{{t}^{2}}$$

Here, $\delta =\frac{1}{{V}_{{\rm{cell}}}}\frac{3}{8}{k}_{{\rm{B}}}\,{N}_{{\rm{A}}}$ with *k*_B_ the Boltzmann constant, *N*_A_ the Avogadro number and ${N}_{m{\rm{pr}}}$ is the number of moles of the products in every irreversible process.

Instead, *r*_*e* exch_ is

5 $${r}_{e{\rm{exch}}}(x,t)=-\eta \,{t}^{\frac{1}{2}}{e}^{\frac{{(x-L)}^{2}}{4\kappa t}}\sum _{k=1}^{{N}_{{\rm{pr}}}}{u}_{k}{e}^{-(|x-L/2|/L+t/\tau )}\frac{{d}_{e}{N}_{mk}}{d{\tau }_{1}}$$

Here, $\eta =\frac{1}{{V}_{{\rm{cell}}}}\frac{1\,}{{x}_{0}}\frac{{(4\pi \kappa )}^{\frac{1}{2}}}{{T}_{0}\,}$, where *x*_0_ is a characteristic length of the order of the size of the normal cell, *T*_0_ is the maximum intercellular temperature, *dτ*_1_ is a characteristic time of the order of the inverse of *k*_kin_, *N*_pr_ is the number of products of the irreversible chemical process with “pr” indicating products and ${d}_{e}{N}_{m}{}_{k}$ is the variation of the number of moles of the products of the irreversible reaction. To derive equation (5) we have substituted the intercellular temperature distribution determined by solving the heat equation in the intercellular environment with no boundary conditions and the expression of the chemical potential. For the detailed calculations leading to equations (4) and (5), respectively see Supplementary Information, Section 3 for the detailed.

As expected, equations (1)–(5) do not fulfil the time-reversal symmetry $(r(x,t)=r(x,-t))$ and this is an elegant demonstration of the irreversible nature of spontaneous processes occurring in living systems. Finally, note that the model developed for a single cell can be extended to a tissue by generalizing equations (1)–(5) to the number of cells composing it and by considering their mutual interactions but this is outside the scope of this work.

### Application of the rate of entropy model to glucose catabolism in normal and cancer breast cells

We have applied the general model, described in the previous section, to glucose catabolism in a typical epithelial breast tissue. In a cell, the glucose catabolism is an essential step in the production of ATP for energy purposes. In general, in differentiated cells there is the production of about 80–90% of the ATP through OXPHOs, while in undifferentiated cells or cancer cells, where there is a prevalence of lactic acid fermentation, this ratio can considerably change and sometimes it can even invert. In the same interval of time the major rapidity of the fermentation process produces a high amount of ATP, as long as a high uptake of glucose and a high expression and activity of lactic acid dehydrogenase, conditions that are present in the predominantly fermentative cellular systems. In addition, there is a major number of processed molecules through the fermentative way in order to maintain a sufficient stock of ATP. In normal cells, the glucose catabolism is 80% through OXPHOs and 20% through lactic acid fermentation, while in cancer cells 90% occurs through lactic acid fermentation and 10% through OXPHOs^20, 21^.

**Figure 1:**
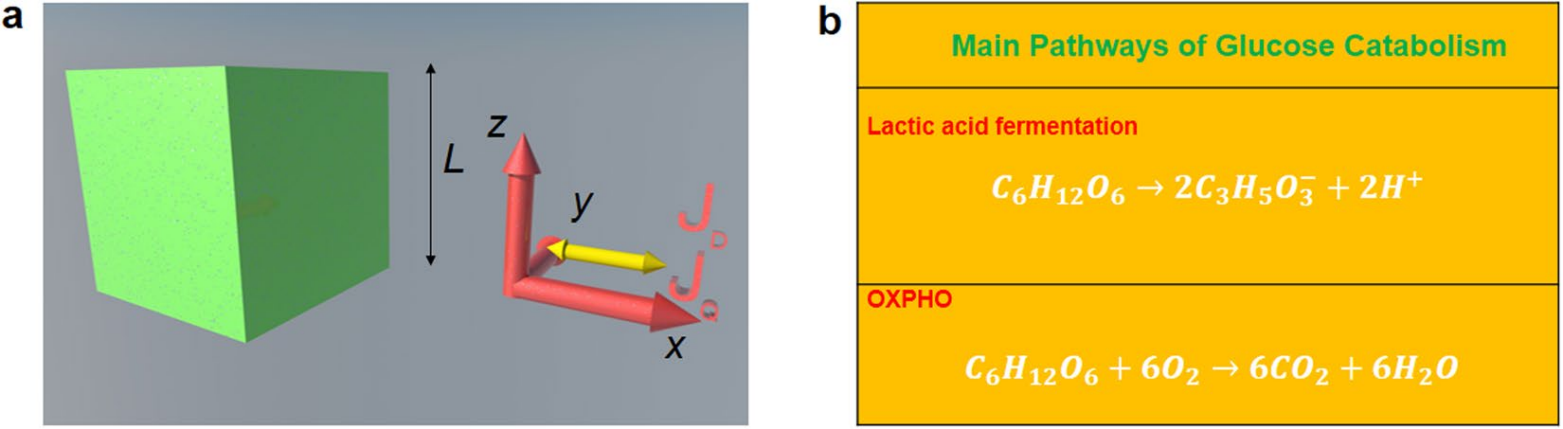
Cell representation and balancing of glucose catabolism. (**a)** Sketch of the cubic cell according to the cyto-morphological features of the epithelial tissue chosen as the reference tissue in our theoretical model. (**b)** Schematics of main pathways of glucose catabolism. The internal heat flow and the mass diffusion flow start in the cytoplasm region for *x* = *L*/2 and are along + *x* and −*x* directions as shown by the yellow arrows.

We show the representative cell (either normal or cancer) in the form of a cubic cell of average side *L* and the glucose catabolism process in Fig. 1 where **J**_*Q*_ is the internal heat flow and **J**_*D*_ is the internal diffusion flow.

In the numerical calculations, we have taken the typical sizes of epithelial cells of human breast tissue (normal or cancer). Specifically, we have employed an average size *L* = 10 *μ*m for the normal cell and *L* = 20 *μ*m for the cancer cell^51^. Moreover, the membrane between the cell and the intercellular environment is of the order of a few Angstroms and it is much smaller than the cell size so that it can be neglected. In addition, for the same tissue we have taken an average size of the intercellular space about 0.2–0.3 *μ*m between two adjacent normal cells and about 1.5 *μ*m between two cancer cells^52^. Within the 1D model employed the flows and the thermodynamic forces generating the flows occur along the *x* direction in the cytoplasm and on both sides. Indeed, according to assumption iii), we have considered that all processes originate in the centre of the cell at *x* = *L*/2 for values of *y* and *z* coordinates corresponding to cytoplasm region.

We distinguish between two kinds of reactions. The first one refers to the cell respiration process involving the catabolism of glucose (C_6_H_12_O_6_) via the oxygen molecule (O_2_) and consists of: (a) glycolysis (b) Krebs cycle and (c) oxidative phosphorylation. The general balancing of all reactions is summarized in the simple form C_6_H_12_O_6_ + 6O_2_ → 6 CO_2 + _6 H_2_O leading to the formation of carbon dioxide (CO_2_) and water (H_2_O). The second process is the lactic acid fermentation process with no Krebs cycle and oxidative phosphorylation. The corresponding reaction leads to the formation of lactic acid ions (C_3_H_5_ O_3_^−^) and is summarized in the simple form C_6_H_12_O_6_ → 2 C_3_H_5_ O_3_^−^ + 2 H^+^. For our purposes, we have not considered the NAD^+^/ NADH and purine nucleotides such as ATP/ADP/AMP^53^ because their concentrations vary within small ranges and do not affect the calculation of the RIEDP and REEDP. Furthermore, the oxide/reduction reactions of NAD^+^/NADH are reversible and the reactions leading to ATP synthesis molecule are not spontaneous and processes irreversible^54, 55^. For the sake of simplicity, note that here we describe the stoichiometric balancing of the above reactions by considering one mole of glucose even though during the glucose catabolism the range of glucose concentration in a single cell is between pico- and micromoles.

## Results

### Numerical calculation of the rate of internal entropy density production for breast cells

**Figure 2:**
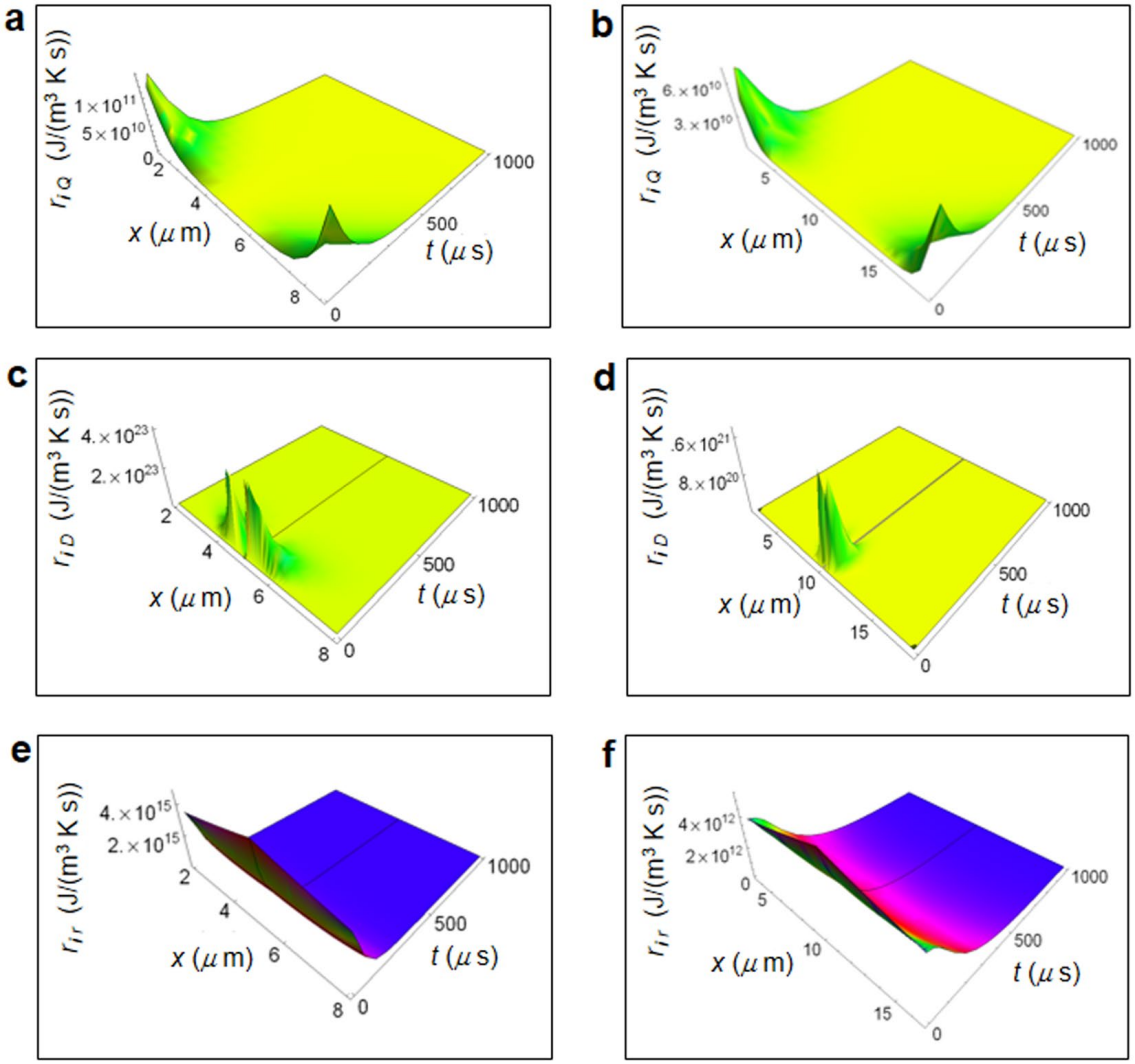
Calculated RIEDP during glucose catabolism for a breast cell. (**a**) RIEDP associated to heat flow for a normal cell. (**b)** RIEDP associated to heat flow for a cancer cell. (**c)** RIEDP related to matter diffusion for a normal cell. (**d)** RIEDP related to matter diffusion for a cancer cell. (**e)** RIEDP due to irreversible reactions for a normal cell. (**f**), RIEDP due to irreversible reactions for a cancer cell.

Figure 2 shows the RIEDP calculated for a normal and cancer breast cell as a function of the spatial coordinate and time^56^ obtained by using the chemical potentials and diffusion coefficients in Tab.1. The use of the chemical potential at standard conditions shown in Table 1 in the numerical calculations is justified by the fact that, within the ideal gas description of a cell, the temperature dependent chemical potential *μ* (*T*) is very close to the *μ* at standard conditions from the Gibbs-Duhem relation being the pressure inside a cell close to the atmospheric pressure.

**Table 1:** Chemical potentials and diffusion coefficients for the chemical species involved in glucose catabolism for cell respiration and lactic acid fermentation. The data are from G. Job and R. Rüffler, Physikalische Chemie, Vieweg + Teubner Verlag Springer (2011).

Chemical species	Chemical potential *μ* (kJ/mole) *T* = 298 K, *p* = 1 atm	Diffusion coefficient *D* in H_2_O (m^2^/s) *T* = 298 K, *p* = 1 atm
C_6_H_12_O_6_	−917.44	6.73 × 10^−10^
O_2_	16.44	21.00 × 10^−10^
CO_2_	−385.99	19.20 × 10^−10^
H_2_O	−237.18	21.00 × 10^−10^
Lactate ion C_3_H_5_O_3_^−^	−516.72	9.00 × 10^−10^
H^+^ aqueous solution	0	45.00 × 10^−10^

We have chosen the time interval of 1000 *μ*s because it a typical internal time^57^ for most biological processes. *r*_i *Q*_, was calculated using equation (1) taking *K* = 0.600 J/(m s K), *κ*_H20_ = 0.143 ×10^−6^ m^2^/s, the thermal diffusivity in water and *p* = 0.85 (0.90) for normal (cancer) cells^21, 38^. *r*_*i D*_ was calculated according to equation (2), while *r*_*i r*_ was calculated according to equation (3) applied to glucose catabolism (see Supplementary Information, Section 2, equations (S17) and (S26), respectively for more details). In addition, *N*_resp_ = 4 (*N*_ferm_ = 3) is the number of chemical species involved in the respiration (fermentation) process, *w*_resp_ = 0.8 (*w*_ferm_ = 0.2) with “resp” (“ferm”) labelling respiration (fermentation) are the corresponding weights of the respiration (fermentation) processes for a normal cell and *w*_resp_ = 0.1 (*w*_ferm_ = 0.9) for a cancer cell^37–39^. We have also taken the pathway kinetic constants ${k}_{{\rm{kin}}}^{{\rm{resp}}}$ = 10^−4^/s and ${k}_{{\rm{kin}}}^{{\rm{ferm}}}$ = 10^−5^/s and both processes, in general, are first-order chemical reactions^57^. In addition, we have used *τ* = 10^−4^ s as typical cell decay time and ${N}_{m{C}_{6}{H}_{12}{O}_{6}}=1$ for both respiration and fermentation processes.

**Figure 3:**
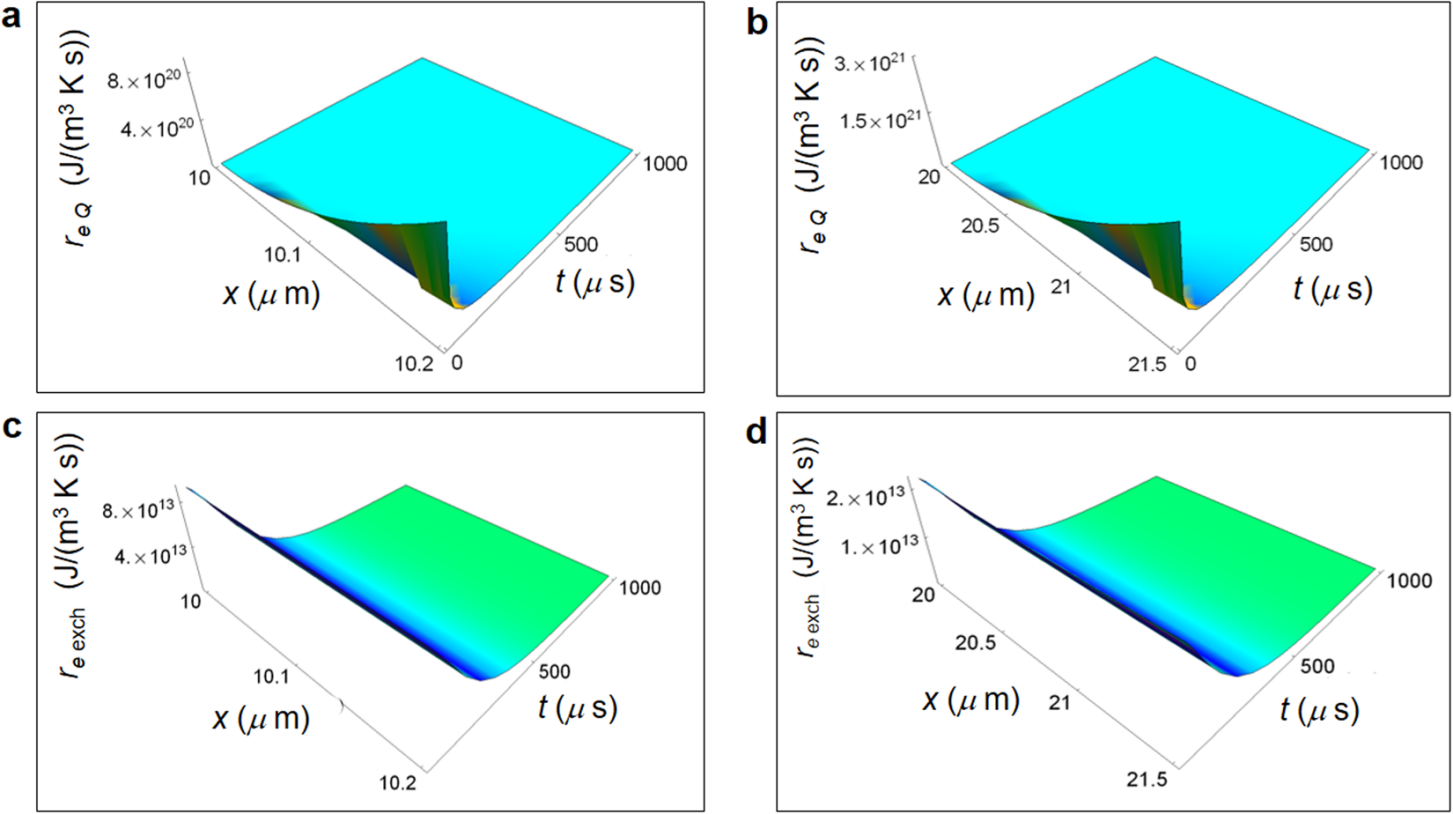
Calculated REEDP during glucose catabolism for a breast cell. (**a)** REEDP associated to heat flow for a normal cell as a function of the spatial and time coordinates. (**b**) REEDP associated to heat flow for a cancer cell. (**c)** REEDP due to exchange of matter for a normal cell. (**d)** REEDP due to exchange of matter for a cancer cell.

*r*_*i Q*_ shown in Fig. 2a and b has a strong variation going towards cell borders and quickly decreases with time. *r*_*i D*_ depicted in Fig. 3c and d exhibits a significant magnitude close to the centre of the cell and strongly decreases towards the border and with increasing time. *r*_*i r*_ displayed in Fig. 2e and f is maximum close to the centre of the cell, slightly reduces the amplitude towards the cell border and strongly decreases with increasing time especially in a normal cell. Both ${r}_{iD}$ and ${r}_{ir}$ are much greater than *r*_*i Q*_ and are about two order of magnitude greater in a normal cell with respect to a cancer cell.

### Numerical calculation of the rate of external entropy density production for breast cells

Figure 3 displays calculated *r*_e *Q*_ and *r*_*e* exch_ for a normal and a cancer breast cell starting from the cell border to the border of the adjacent cell taking into account the typical size of the intercellular space for this tissue, namely about 0.2 – 0.3 *μ*m for a normal cell and about 1.5 *μ*m for a cancer cell^52^.

*r*_*e Q*_ was calculated according to equation (4) applied to glucose catabolism, while *r*_*e* exch_ was calculated according to equation (5) applied to glucose catabolism (see Supplementary information, Section 3, equation (S32) and equation (S36), respectively). The parameters used in the numerical calculations are *k*_B_ = 1.3805 × 10^−23^ J/K, *N*_*m*pr resp_ = 12, the number of moles of the products of respiration, *N*_*m*pr resp_ = 4, the number of moles of the lactic acid fermentation reaction. In addition, like for the calculation of the rate of internal entropy density production we have taken *w*_resp_ = 0.8 (*w*_ferm_ = 0.2), the corresponding weights of the respiration (fermentation) processes for a normal cell and *w*_resp_ = 0.1 (*w*_ferm_ = 0.9) for a cancer cell^37–39^. The trend of *r*_*e Q*_ and *r*_*e* exch_ for cancer cells is suitable to the observations^29, 30, 32^.

As shown in Fig. 3a and b, *r*_*e Q*_ increases as a function of *x* for vanishing time and decreases with increasing time. *r*_*e* exch_ displayed in Fig. 3c and d does not vary appreciably as a function of *x* and decreases with increasing time. The magnitude of *r*_*e Q*_ is much larger than the corresponding *r*_*e* exch_ and this behaviour is enhanced in cancer cells where *r*_e *Q*_ is about one order of magnitude larger than in normal cells.

## Discussion

According to the described model, we can draw important conclusions about the cell behaviour during glucose catabolism in terms of entropy production. While *r*_*i*_ is mainly due to matter exchanges, the greatest contribution to *r*_*e*_ comes from the heat irreversible release. Moreover, the amount of *r*_*e*_ is greater for a cancer cell and, due to the minor volume of a normal cell, *r*_*i*_ is on average greater for a normal cell. In contrast, *r*_*e*_ is greater in a cancer cell due to the larger contribution of *r*_*e Q*_ because of the prevalence of the dependence on the spatial coordinate with respect to that on the cell volume.

Note that we have applied the model to the particular case of cells belonging to breast tissue but it would be still valid if applied to different tissues characterized by other volume ratios between normal and cancer cells and easily extended to staminal cells that are of much smaller size.

As shown in Figs 2 and 3 for the epithelium breast normal and cancer cells, we have proved that $r(x,t)={r}_{i}(x,t)+{r}_{e}(x,t)$ tends towards a minimum value with increasing time such that the cell reaches a state of global equilibrium in accordance with the minimum dissipation Prigogine’s theorem. Moreover, looking especially at Fig. 2 displaying $\,{r}_{i}(x,t)$, the rate of entropy density production tends to its minimum value more slowly in a cancer cell with respect to a normal cell. This is a key point because it demonstrates that the tendency to stability or equivalently to global equilibrium is slower in a cancer cell. We can easily prove the fulfilment of the minimum dissipation Prigogine’s theorem calculating $r(x,t)$ for the other irreversible reactions via equations (1–5). So far, there has been an application of this theorem to the uptake of the oxygen molecule in cells that is proportional to the released heat. However, albeit partially correct, this application does not take into account the weight of glycolysis and of lactic acid fermentation or of other chemical reactions involving oxygen consumption. Instead, our model overcomes these limits encompassing quantitatively all those aspects and is not restricted to oxygen molecule consumption. We emphasize that the minimum dissipation of entropy is necessary to establish a condition compatible with the behaviour of Gibbs free energy in living systems because of the thermodynamic relation among free energy, enthalpy and entropy.

At the same time, we believe that the model, although giving general relations, represents a starting point and the results obtained are only an indication of what could occur in a specific calculation. Moreover, note that the calculated rates of entropy are not purely theoretical but strongly depend on consolidated experimental data like, for example, the frequency of occurrence of an irreversible process, the cellular volume and the average intercellular size. We wish that some experiments along this direction will be carried out to verify the importance of the calculations because we believe that measurements of the rate of entropy could bring new fundamental knowledge in the field shedding light on the important role played by metabolic, epigenetic and gene networks in living systems. For example, the number of moles of chemical species appearing in the main pathways like OXPHO and lactic acid fermentation can be carefully measured, e.g *in vitro*/*in vivo* via imaging techniques by means of Nuclear Magnetic Resonance or Positive Emission Tomography (PET). This leads to a quantitative estimate of the rate of entropy due to mass transport opening the root for new measurements in cell biology. Note that the model can be applied to surfaces of internal parenchyma generalizing the rate of entropy relations found for a single cell to agglomerates of cells displayed with PET technique. On the other hand, measurements done by means of microcalorimetry technique may be employed to quantify the heat flow.

As the glucose catabolism is strongly correlated with the development of cancer and self-renewal processes, the described model, if validated by specific and real measures of glucose absorption^29, 30^ could introduce a new way to quantify the glucose catabolism in relation to the exchange of entropy occurring during this irreversible process.

Moreover, the interplay between the glucose uptake or production of lactate ion and the rate of entropy might be particularly important for clinical studies on cancer development and self-renewal process^58^. It is also interesting to note that the developed formalism might be used also for electrophysiological processes with special regard to ionic fluxes. These processes have a crucial role in the development and progression of neoplastic processes and, in addition, the modulation of ionic currents might modulate the patterns of metabolic network^42, 59^.

In summary, although the thermodynamics of irreversible processes introduced by De Donder and subsequently developed by Prigogine is, often, regarded as mainly theoretical and speculative, it could play a crucial role in describing a unified phenomenological paradigm of biological systems. More precisely, when modern biology faces the challenge of a systemic vision of life in terms of interactions among complex networks, the study of the rate of entropy represents a starting point able to reveal the phenomenological and unitary feature of life. Indeed, our results show that the study of the rate of entropy density production open new perspectives in modern biology giving a simple picture of irreversible processes in living systems.

## Methods

### Basic principles

Let us consider an open system (cell) that exchanges energy and matter with the environment. For a system in local equilibrium but not in global equilibrium, it is convenient to define an entropy density $s=S/{V}_{{\rm{cell}}}$ with $s={s}_{i}+{s}_{e}$ where ${s}_{i}$ is the internal entropy density (subscript “*i*” indicates internal), ${s}_{e}$ the external entropy density (subscript “*e*” indicates external) and ${V}_{{\rm{cell}}}$ the volume of the cell. The entropy density infinitesimal variation $ds=d{s}_{i}+d{s}_{e}$ includes the contribution *ds*_*i*_ related to internal irreversible processes and *ds*_*e*_ associated to the external exchanges of heat and matter with the intercellular environment. To fully describe the temporal evolution, it is useful to define the rate of entropy density production in a time interval *dt* in the form $r=d{s}_{i}/dt+d{s}_{e}/dt$. This quantity gives a direct measure of irreversible processes.

### Rate of internal entropy density production

First, we express in a time interval $dt$ the rate of internal entropy density production ${r}_{i}=\frac{d{s}_{i}}{dt}$ giving the amount of local increase of entropy inside a cell that can be regarded as a continuous system. In a compact form^43^

6 $${{r}}_{{i}}\,({\bf{x}}{,}t){\boldsymbol{=}}{\boldsymbol{\nabla }}(\frac{{\rm{1}}}{{T}({\bf{x}}{,}t)})\cdot {{\bf{J}}}_{u}\,({\bf{x}}{,}t)-\sum _{k=1}^{N}{\boldsymbol{\nabla }}(\frac{{\mu }_{k}({\bf{x}},t)}{{T}({\bf{x}}{,}t)})\cdot {{\bf{J}}}_{Dk}\,({\bf{x}}{,}t)+\frac{1}{{T}({\bf{x}}{,}t)}\sum _{j=1}^{M}{A}_{j}({\bf{x}}{,}t)\,{{\rm{v}}}_{j}$$

Here, the first term on the second member is associated with the internal flow ${{\bf{J}}}_{u}{\boldsymbol{=}}{{\bf{J}}}_{Q}+\sum _{k=1}^{N}{{u}}_{{k}}{{\bf{J}}}_{Dk}$, with *N* the number of chemical species, getting contributions from irreversible heat flow ${{\bf{J}}}_{Q}$ and diffusion flow ${{\bf{J}}}_{Dk}$ and driven by the heat thermodynamic force ${{\bf{F}}}_{u}({\bf{x}}{,}t){\boldsymbol{=}}{\boldsymbol{\nabla }}{({T}({\bf{x}}{,}t))}^{-1}$. The second term is related to the diffusion flow ${{\bf{J}}}_{Dk}$ and is driven by the matter thermodynamic force ${{\bf{F}}}_{k}({\bf{x}}{,}t){\boldsymbol{=}}{\boldsymbol{\nabla }}(\frac{{\mu }_{k}({\bf{x}},t)}{{T}({\bf{x}}{,}t)})$ with ${\mu }_{k}$ the chemical potential of the *k*th chemical species.

Finally, the last term is associated with irreversible reactions being *M* the number of chemical reactions with the thermodynamic force represented by the affinity ${A}_{j}({\bf{x}},t){\boldsymbol{=}}{\boldsymbol{-}}\sum _{k=1}^{N}{\nu }_{jk}{\mu }_{k}({\bf{x}},t)$ with ${\nu }_{jk}$ the stoichiometric coefficients, and the corresponding flow by the velocity of the *j*_th_ reaction ${{\rm{v}}}_{j}=\frac{1}{V{\rm{cell}}}\frac{d{\xi }_{j}}{dt}$ with $d{\xi }_{j}$ the variation of the *j*th degree of advancement.

### Rate of external entropy density production

We express, in the same time interval $dt$ the rate of external entropy density production ${r}_{e}=\frac{d{s}_{e}}{dt}$ giving the amount of local increase of entropy outside the cell corresponding to the intercellular environment. In its general form

7 $${r}_{e}\,({\bf{x}},t){\boldsymbol{=}}\frac{1}{{T}_{{\rm{i}}{\rm{c}}}\,({\bf{x}},t)}\frac{1}{{V}_{{\rm{c}}{\rm{e}}{\rm{l}}{\rm{l}}}}\frac{dQ}{dt}-\frac{1}{{T}_{{\rm{i}}{\rm{c}}}\,({\bf{x}},t)}\sum _{k=1}^{{{\boldsymbol{N}}}_{{\rm{p}}{\rm{r}}}}{\mu }_{k}({\bf{x}},t)\,\frac{{d}_{e}{N}_{mk}}{dt}$$

here, $\frac{1}{{V}_{{\rm{cell}}}}\frac{dQ}{dt}=\frac{du}{dt}+\frac{1}{{V}_{{\rm{cell}}}}p\frac{dV}{dt}$ according to the first principle of thermodynamics. $dQ=dU+pdV$ with $dQ$ the infinitesimal heat transfer,$du=\frac{1}{V}dU$ the infinitesimal variation of the internal energy density *u* (d*U* is the infinitesimal variation of the internal energy *U*), *p* the pressure and *dV* the infinitesimal variation of the volume *V*. In addition, ${T}_{{\rm{ic}}}\,({\bf{x}}{,}t)$ is the intercellular temperature distribution and *d*_*e*_ *N*_*m k*_ the variation of the number of moles of the *k*th chemical species with ${{N}}_{{\rm{pr}}}$ the number of products of the irreversible reaction (“pr” indicates products) due to exchange of matter with the intercellular environment.

The first term on the second member expresses the heat flow outside the cell, while the second one results from the exchange of matter with the intercellular environment.

### Diffusion equations

In order to find the heat and mass flow appearing in the expression of the RIEDP given in equations (1) and (2), we employ the well-known heat and mass transport equations. We consider a rectangular frame *xyz* with the origin in the center of the cell and we suppose that the flow direction is along *x* (see Fig. 1).

Concerning the heat diffusion, it is mainly due to a conduction transport neglecting the convection transport present to a much lesser extent inside a typical cell and the term of heat source. Hence, it is

8 $$\frac{\partial T({\bf{x}},t)}{\partial t}=\kappa {\nabla }^{2}T({\bf{x}},t)$$

where the solution *T*(**x**,*t*) is the temperature distribution function depending on both spatial and time variable, $\kappa =\frac{K}{{c}_{{\rm{s}}}\rho }$ is the thermal diffusivity (m^2^ s^−1^) with *K* the thermal conductivity expressed in J m^−1^ s^−1^ K^−1^) and supposed uniform, *c*_s_ the specific heat expressed in J Kg^−1^ K^−1^ and *ρ* the density expressed in Kg m^−3^. The flow ${J}_{Q}({\bf{x}},t)$ is proportional to the spatial derivative of $\,T({\bf{x}},t)$.

Analogously, the mass transport equation or Fick’s second diffusion law reads

9 $$\frac{\partial n({\bf{x}},t)}{\partial t}=D{\nabla }^{2}n({\bf{x}},t)$$

where, the solution $n({\bf{x}},t)$ is the concentration of molecules, a distribution function depending both on spatial and time variables and *D* is the diffusion coefficient (m^2^ s^−1^) supposed uniform. The flow ${J}_{D}\,({\bf{x}},t)$ is proportional to the spatial derivative of $n\,({\bf{x}},t)$.

## Electronic supplementary material

Supplementary Information
