# Supplementary material for: Rate of entropy model for irreversible processes in living systems
Source: Sci Rep. 2017 Aug 22;7:9134. doi: 10.1038/s41598-017-09530-5 (PMC5567375; doi:10.1038/s41598-017-09530-5)
Supplement: Supplementary file 1 — Supplementary Information [file 41598_2017_9530_MOESM1_ESM.pdf]

SUPPLEMENTARY INFORMATION

Rate of entropy model for irreversible processes in living systems

R. Zivieri[1,2], N. Pacini[3], G. Finocchio[2], M. Carpentieri[4]

[1]*Department of Physics and Earth Sciences and Consorzio Nazionale Interuniversitario per le Scienze Fisiche della Materia, Unit of Ferrara, University of Ferrara, via G. Saragat 1, Ferrara I-44122, Italy*

[2]*Department of Mathematical and Computer Sciences, Physical Sciences and Earth Sciences, University of Messina, V.le F. D'Alcontres, 31, 98166, Messina, Italy*

[3]*Department of General Surgery, Section of Senology, University Hospital Company, Policlinico Vittorio Emanuele, via S. Citelli 6, 95124, Catania, Italy*

[4]*Department of Electrical and Information Engineering, Politecnico di Bari, via E. Orabona 4, Bari I-70125, Italy*

1. OVERVIEW

We restrict ourselves to the study of the thermodynamics in the single cell representing an open thermodynamic system. Since we are dealing with phenomena taking place locally and under conditions of local equilibrium inside and outside a typical cell, all intensive and extensive thermodynamic variables have a space and time dependence [S1]. On this basis, it is useful to define the rate of entropy density production, $r = r_i + r_e$, where r_i is the rate of internal entropy density production (RIEDP) and r_e the rate of external entropy density production (REEDP). Starting from the definition of the entropy density $s = \frac{S}{V_{\text{cell}}}$ with $s = s_i + s_e$ where s_i is the internal entropy density, s_e the external entropy density and V_{cell} the volume of the cell it is possible to compute *r*.

In the next two sections, we give the details about the calculation of r_i and r_e, respectively valid for any irreversible processes occurring in a cell, either normal or cancer. The calculation lies on thermodynamic arguments combined with heat and mass transport equations. The expressions are general and valid for any irreversible chemical process and we then apply them to glucose catabolism.

To calculate the rate of entropy density production, for the sake of convenience and without loss of generality, we represent the cell (either normal or cancer) as a cube of volume $V_{\text{cell}} = L^3$ taking as reference the breast epithelium tissue. Here, *L* is the average size with $L = 10$ μm for a typical normal cell and $L = 20$ μm for a cancer cell [S2] and we assume that the flows occur mainly along the *x* direction within a 1D model (see Fig. 1 in the main text). In the numerical calculations shown in the following we assume that all irreversible processes take place at $x = L/2$ and for values of *y* and *z* corresponding to the region of the cytoplasm where glucose catabolism occurs (see the main text for more details).

To describe the glucose catabolism we recall the two reactions described in detail in the main text, namely the respiration process and the lactic acid fermentation process involving glucose ($C_6H_{12}O_6$) catabolism. The respiration process is summarized as $C_6H_{12}O_6 + 6O_2 \rightarrow 6\ CO_2 + 6\ H_2O$ leading to the formation of carbon dioxide (CO_2) and water (H_2O). The lactic acid fermentation process leads to the formation of two lactic acid ions ($C_3H_5O_3$-) and two protons (H^+) and is summarized in the

simple form $C_6H_{12}O_6 \rightarrow 2\, C_3H_5O_3^- + 2\, H^+$ [S3, S4] (for a more detailed discussion on this point see the main text).

2. RATE OF INTERNAL ENTROPY DENSITY PRODUCTION

We define the space and time dependent RIEDP $r_i(\mathbf{x},t) = \frac{ds_i(\mathbf{x},t)}{dt}$ (with $\mathbf{x} = (x, y, z)$ and t the time) giving the amount of local increase of entropy in continuous thermodynamic systems. In our special case, we consider a cell (either normal or cancer) and the RIEDP associated to irreversible processes occurring inside it.

In order to do that, we recall its general expression in terms of heat flow and mass flow (see Methods):

$$r_i(\mathbf{x},t) = \nabla\left(\frac{1}{T(\mathbf{x},t)}\right) \bullet \mathbf{J}_u(\mathbf{x},t) - \sum_{k=1}^{N} \nabla\left(\frac{\mu_k(\mathbf{x},t)}{T(\mathbf{x},t)}\right) \bullet \mathbf{J}_{D\,k}(\mathbf{x},t) + \frac{1}{T(\mathbf{x},t)} \sum_{j=1}^{M} A_j(\mathbf{x},t)\, \mathrm{v}_j \qquad \text{(S1)}$$

Here, $\nabla\left(\frac{1}{T(\mathbf{x},t)}\right)$ is the thermodynamic force leading to the internal energy flow $\mathbf{J}_u = \mathbf{J}_Q + \sum_{k=1}^{N} u_k \mathbf{J}_{D\,k}$ with $\mathbf{J}_Q$ the heat flow, u_k the partial molar energy and $\mathbf{J}_{D\,k}$ the diffusion flow of the kth chemical species with N the number of chemical species. In particular, $u_k = \left(\frac{\partial u}{\partial n_{m\,k}}\right)_T$ is expressed in J/mole with u the energy density expressed in J/m^3, $n_{m\,k} = \frac{N_{m\,k}}{V}$ the number of moles per unit volume of the kth species with $N_{m\,k}$ the number of moles. Instead, $\nabla\left(\frac{\mu_k(\mathbf{x},t)}{T(\mathbf{x},t)}\right)$ is the kth thermodynamic force giving rise to the diffusion flow $\mathbf{J}_{D\,k}(\mathbf{x},t)$. Finally, $A_j(\mathbf{x},t)$ is the affinity of the jth chemical reaction and v_j is the velocity of reaction with M the number of chemical reactions.

It is possible to identify three different contributions to the RIEDP: 1) the one associated to heat transport; 2) the one related to molecules diffusion and 3) the one due to irreversible chemical reactions. The second and third contributions are those due to mass transport. The heat transport is

caused by the temperature gradient inside the human cell. Within this model, we do not include the term $\nabla\left(\frac{1}{T(\mathbf{x},t)}\right)\bullet\sum_{k=1}^{N}u_k\mathbf{J}_{Dk}(\mathbf{x},t)$ that expresses the thermodynamic force due to temperature distribution gradient inside the cell and the corresponding mass flow. In this way, we do not take into account the contribution to the rate of internal entropy density caused by diffusion of chemical species whose flow results from the temperature distribution gradient.

A. RATE OF INTERNAL ENTROPY PRODUCTION ASSOCIATED TO HEAT TRANSPORT

First, we derive the RIEDP associated to heat transport related to the flow $\mathbf{J}_Q(\mathbf{x},t)$ within our model. In its general form:

$$r_{iQ}(\mathbf{x},t)=\mathbf{F}_Q(\mathbf{x},t)\cdot\mathbf{J}_Q(\mathbf{x},t) \tag{S2}$$

Here, $\mathbf{F}_Q(\mathbf{x},t)=\nabla\left(\frac{1}{T(\mathbf{x},t)}\right)$ is the thermodynamic force. In our 1D model, without loss of generality, flows are assumed along *x*; hence, $\mathbf{x}=(x,0,0)$, $\nabla\rightarrow\frac{\partial}{\partial x}\hat{i}$, $T(\mathbf{x},t)\rightarrow T(x,t)$ and $\mathbf{J}_Q=(J_Q,0,0)$. Heat flow occurs symmetrically with respect to $x = L/2$ along the two directions (*x* and –*x*).

To calculate the heat flow, we make the assumption that heat diffusion is mainly due to a conduction transport neglecting, in a first approximation, the convection transport present to a much lesser extent inside a typical cell. The heat transport equation in the 1D case, neglecting the term of heat source, takes the well-known form:

$$\frac{\partial T(x,t)}{\partial t}=\kappa\frac{\partial^2 T(x,t)}{\partial x^2} \tag{S3}$$

Here, the solution to Equation (S3) $T(x,t)$ is the temperature distribution function depending both on spatial and time variable, and, for a given material, $\kappa = K/(c_S\rho)$ is the thermal diffusivity in m^2 /s with K the thermal conductivity in J/ (m s K), c_S the specific heat in J /(Kg K) and ρ the density in Kg/

m^3. Both K and κ are assumed uniform throughout the cell. We impose the following initial and boundary conditions on the temperature distribution:

$$\begin{cases} T\left(x,0\right)=T_0 \ \text{ for } 0\le x\le L & \text{initial condition} \\ T\left(0,t\right)=T\left(L,t\right)=0 \ \text{ for } t>0 & \text{boundary conditions} \end{cases} \tag{S4}$$

This choice is not restrictive because the temperature vanishes only exactly at the cell border due to the boundary conditions corresponding to the cell membrane and has a weak dependence on x inside the cell. However, note that the temperature on the cell membrane is not zero as inferred from the expression of the temperature distribution in the intercellular environment (see paragraph B for details) obtained in the absence of boundary conditions. The solution to equation (S3) taking into account the initial and boundary conditions expressed by equation (S4) reads

$$T\left(x,t\right)=\frac{4T_0}{\pi}\sum_{n=1}^{\infty}\left(\frac{\sin\left[\left(2n-1\right)\frac{\pi}{L}x\right]}{2n-1}e^{-\kappa\left(2n-1\right)^2\frac{\pi^2}{L^2}t}\right) \tag{S5}$$

A graphical solution to equations (S4) and (S5) of the temperature distribution (in Kelvin, K) inside the cell is shown in Fig. S1a and Fig.S1b as a function of x and of t with t ranging from 0 to 1000 μs, a typical cell time interval. In the numerical calculations, we have taken the initial temperature T_0 = 310 K and, taking into account that the cytoplasm is mainly composed by water, we have used the thermal diffusivity of water, viz. κ_{H20} = 0.143 $\times 10^{-6}$ m^2/s. The temperature is almost uniform and, on average, about T_0 especially for the initial instant of times (see Fig.S1c and Fig.S1d). The temperature vanishes only at the border corresponding to the cellular membrane for $t > 0$ because of the boundary condition of equation (S4).

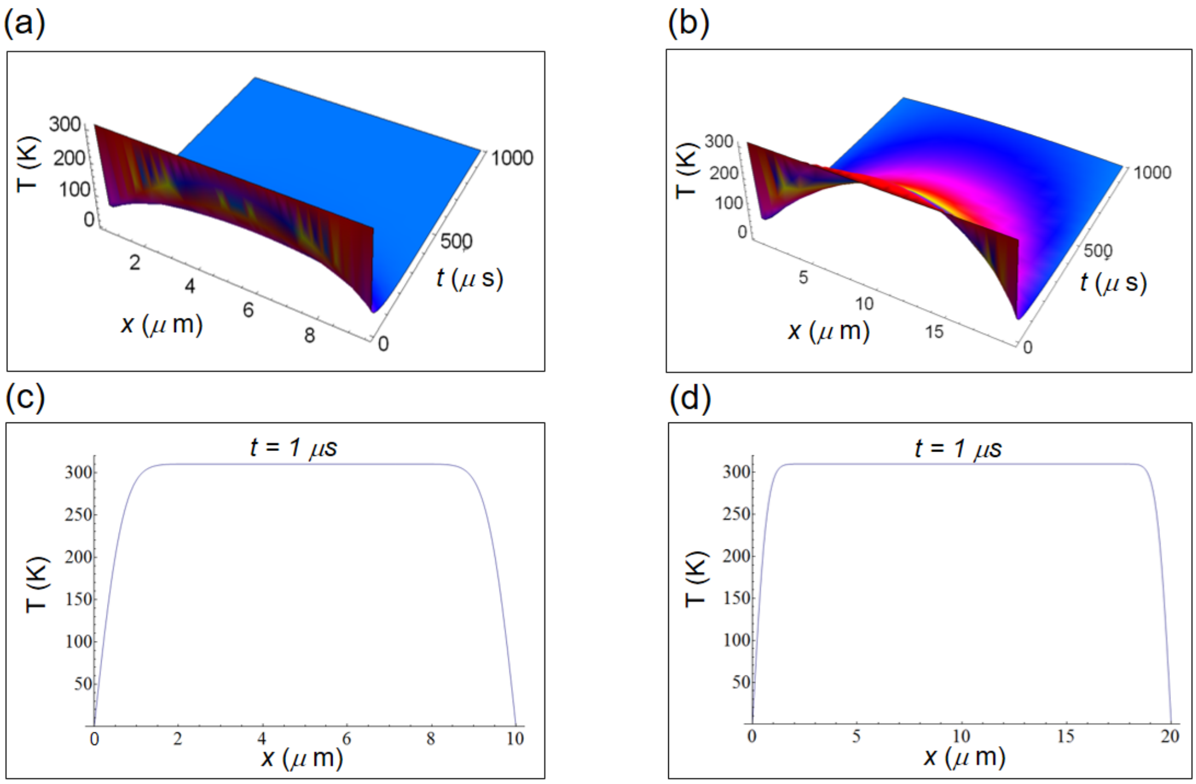

Figure S1. Temperature distribution inside a human cell according to equations (S4) and (S5). (a) Temperature distribution inside a normal cell as a function of the spatial coordinate and time. (b) Temperature distribution inside a cancer cell as a function of the spatial coordinate and time. (c) Temperature as a function of the spatial coordinate in a normal cell. (d) Temperature as a function of the spatial coordinate in a cancer cell.

For the 1D case $\mathbf{F}_Q = \left(F_Q, 0, 0\right)$ with $\mathbf{F}_Q\left(x,t\right) = \frac{\partial}{\partial x}\left[\left(T\left(x,t\right)\right)^{-1}\right] e^{-\frac{t}{\tau}}\,\hat{\imath}$ expressed in 1/ (m K) where an exponential time decay depending on a typical decay time τ has been included to describe the time evolution of the force. Hence, by inserting the solution to heat equation given in equation (S5), we get

$$\mathbf{F}_Q\left(x,t\right) = -\frac{\pi^2}{4}\frac{1}{T_0}\frac{1}{L}\frac{\sum_{n=1}^{\infty}\left(\cos\left[\left(2n-1\right)\frac{\pi}{L}x\right]e^{-\kappa(2n-1)^2\frac{\pi^2}{L^2}t}\right)e^{-\frac{t}{\tau}}}{\left(\sum_{n=1}^{\infty}\frac{1}{2n-1}\left(\sin\left[\left(2n-1\right)\frac{\pi}{L}x\right]e^{-\kappa(2n-1)^2\frac{\pi^2}{L^2}t}\right)\right)^2}\,\hat{\imath} \qquad \text{(S6)}$$

Fig. S2 displays the force expressed in equation (S6) for a normal and a cancer cell with $\tau = 10^{-4}$ s a typical cell decay time. In the interval $0 \le x \le L/2$ we have taken the modulus of $\mathbf{F}_Q$ to have the force positive in the whole interval. The force becomes greater passing from the center to the border and reduces its magnitude with increasing time. This force is only due to the internal temperature distribution and it is the same for all chemical species.

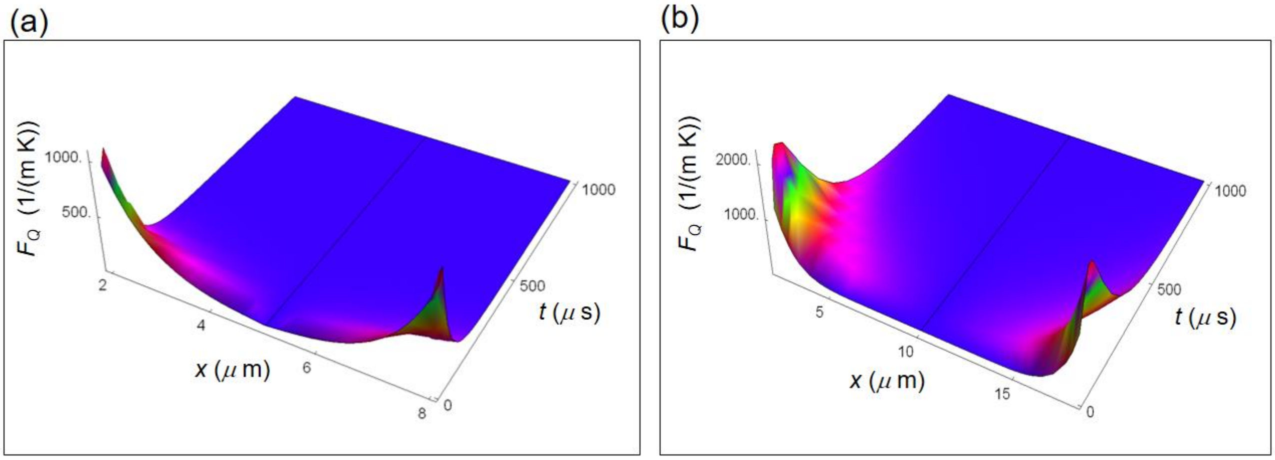

Figure S2. Force associated to internal heat flow calculated by means of equation (S6) as a function of the spatial coordinate and of time. (a) Force inside a normal cell. (b) Force inside a cancer cell.

Within the 1D model, the heat flow per unit time and area expressed in J/ (m^2 s) is $\mathbf{J}_Q = \left(J_Q, 0, 0\right)$ with $J_Q = \frac{1}{A}\frac{dX_Q}{dt}$, dX_Q the heat flow and A is the cell area. According to Fourier law, the heat flow per unit time and per unit area along x is $J_Q(x,t) = -K\frac{\partial T(x,t)}{\partial x}$. The minus sign on the second member only indicates that heat flows from the region at higher temperature corresponding to the cell centre to the region at lower temperature close to the cell membrane, namely in the direction along which the temperature decreases (in this case it is symmetrical along $+x$ and $-x$). Explicitly

$$\mathbf{J}_Q\left(x,t\right) = -\,4\;p\;K\;T_0\,\frac{1}{L}\sum_{n=1}^{\infty}\left[\cos\left[\left(2n-1\right)\frac{\pi}{L}x\right]e^{-\kappa\left(2n-1\right)^2\frac{\pi^2}{L^2}t}\right]\hat{i} \qquad \text{(S7)}$$

Here, p denotes the frequency of occurrence of the irreversible reaction. In Fig. S3, we display the bidirectional heat flow per unit time and area given in equation (S7) for both the normal and the cancer cell referred to glucose catabolism. For $0 \le x \le L/2$ we have plotted the modulus of the heat flow to have J_Q positive in the whole interval. In the numerical calculations we have taken $K = 0.600$

J/ (m s K) and, for the case of glucose catabolism, p = 0.90 for cancer cells and p = 0.85 for normal cells [S5] (see the main text for more details). The trend of J_Q is symmetric with respect to the centre for the two types of cells, is less sharp for a cancer cell especially for t ranging between 0 and 100 μs and tends to zero with increasing time.

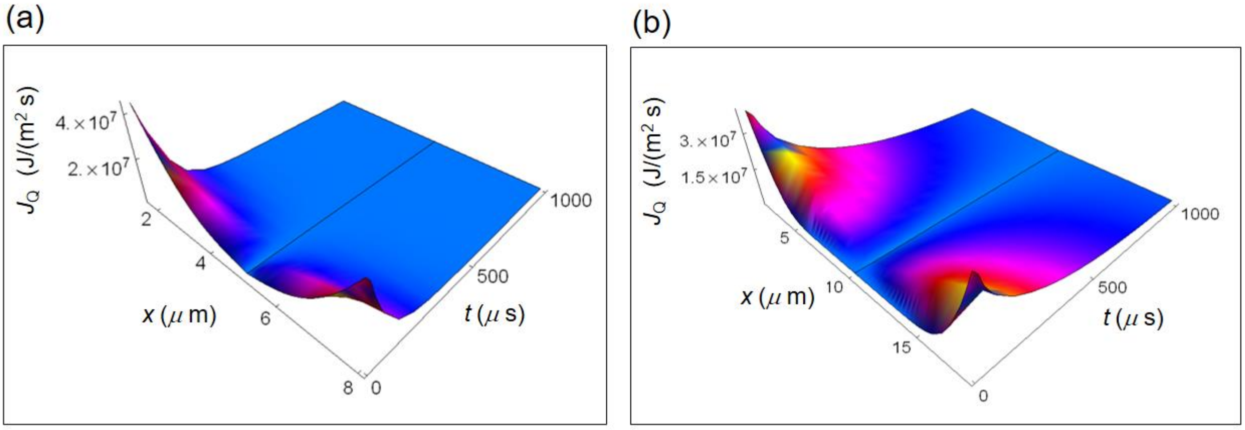

Figure S3. Internal heat flow per unit time and area calculated according to equation (S7) plotted as a function of the spatial coordinate and of time. (a) Heat flow inside a normal cell. (b) Heat flow inside a cancer cell.

The RIEDP due to the heat flow and expressed in J/ (m^3 K s), via equation (S6) and equation (S7), is

$$r_{i\,Q}(x,t) = p\,K\,\frac{\pi^2}{L^2}\,\frac{\left[\sum_{n=1}^{\infty}\left(\cos\left[(2n-1)\frac{\pi}{L}x\right]e^{-\kappa(2n-1)^2\frac{\pi^2}{L^2}t}\right)\right]^2 e^{-\frac{t}{\tau}}}{\left[\sum_{n=1}^{\infty}\left(\frac{1}{2n-1}\sin\left[(2n-1)\frac{\pi}{L}x\right]e^{-\kappa(2n-1)^2\frac{\pi^2}{L^2}t}\right)\right]^2} \qquad \text{(S8)}$$

From equation (S8), it turns out that $r_{iQ}(x,t) \geq 0$. Equation (S8) is equation (1) of the main text.

Note that both the numerator and the denominator can be written in terms of the product of two identical series, namely

$$\left[\sum_{n=1}^{\infty}\left(\cos\left[(2n-1)\frac{\pi}{L}x\right]e^{-\kappa(2n-1)^2\frac{\pi^2}{L^2}t}\right)\right]^2=\sum_{n=1}^{\infty}\left(\cos\left[(2n-1)\frac{\pi}{L}x\right]e^{-\kappa(2n-1)^2\frac{\pi^2}{L^2}t}\right)\times\sum_{n=1}^{\infty}\left(\cos\left[(2n-1)\frac{\pi}{L}x\right]e^{-\kappa(2n-1)^2\frac{\pi^2}{L^2}t}\right)$$

$$\left[\sum_{n=1}^{\infty}\left(\frac{1}{2n-1}\sin\left[(2n-1)\frac{\pi}{L}x\right]e^{-\kappa(2n-1)^2\frac{\pi^2}{L^2}t}\right)\right]^2=\sum_{n=1}^{\infty}\left(\frac{1}{2n-1}\sin\left[(2n-1)\frac{\pi}{L}x\right]e^{-\kappa(2n-1)^2\frac{\pi^2}{L^2}t}\right)\times\sum_{n=1}^{\infty}\left(\frac{1}{2n-1}\sin\left[(2n-1)\frac{\pi}{L}x\right]e^{-\kappa(2n-1)^2\frac{\pi^2}{L^2}t}\right) \quad (S9)$$

B. RATE OF INTERNAL ENTROPY DENSITY PRODUCTION DUE TO DIFFUSION PROCESS

From equation (S1), we express the contribution to RIEDP related to diffusion process via the term

$$r_{i\,D}(\mathbf{x},t) = -\sum_{k=1}^{N}\mathbf{F}_k^{(2)}(\mathbf{x},t)\bullet\mathbf{J}_{D\,k}(\mathbf{x},t) \quad (S10)$$

The thermodynamic force that gives rise to matter (chemical species) flow is $\mathbf{F}_k^{(2)}(\mathbf{x},t)=\nabla\left(\frac{\mu_k(\mathbf{x},t)}{T(\mathbf{x},t)}\right)$. $\mathbf{F}_k^{(2)}(\mathbf{x},t)$ is a new force depending on the chemical species *k* that determines diffusion flow and is expressed in J/(mole m K). Within this description in terms of densities, in chemical reactions $u_k=\left(\frac{\partial u}{\partial n_{m\,k}}\right)_T$ is the partial molar energy of the *k*th chemical species expressed in J/mole with *u* the energy density expressed in J/m^3, $n_{\mathrm{m}\,k} = N_{\mathrm{m}\,k}/V$ the number of moles per unit volume of the *k*th species with $N_{\mathrm{m}\,k}$ the number of moles. Instead, the chemical potential of the *k*th chemical species is determined as $\mu_k(\mathbf{x},t)=\left(\frac{\partial g(\mathbf{x},t)}{\partial n_{m\,k}}\right)_{T,P}$ where $g = G/V$ is the Gibbs free energy density at constant pressure and *G* is the Gibbs free energy. According to its general definition given above, the chemical potential depends on the temperature and on the pressure.

For the 1D case, $\mathbf{F}_{\mathrm{k}}^{(2)}=\left(F_{\mathrm{k}}^{(2)},0,0\right)$ with $\mathbf{F}_k^{(2)}=\frac{\partial}{\partial x}\left(\frac{\mu_k(x,t)}{T(x,t)}\right)\hat{i}$. Here, $\mu_k(x,t)=u_k e^{-(|x-L/2|/L+t/\tau)}$ is the time and space dependent chemical potential of the *k*th chemical species with τ a typical cell decay time; u_k is the chemical potential calculated at $x = L/2$, where it is assumed that the glucose catabolism takes place, that is equal to the partial molar energy. According to this form, the chemical

potential decreases passing from the center to the border of the cell and decreases with increasing time. For every irreversible reaction and for every chemical species $\mathbf{F}_k^{(2)}\ (x,t)$ is

$$\mathbf{F}_k^{(2)}(x,t) = -\frac{\pi^2}{4}\frac{1}{T_0}\frac{1}{L}\times$$
$$\left(\frac{u_k e^{-(|x-L/2|/L+t/\tau)}\left(\pm\frac{1}{\pi}\sum_{n=1}^{\infty}\left(\frac{1}{2n-1}\sin\left[(2n-1)\frac{\pi}{L}x\right]e^{-\kappa(2n-1)^2\frac{\pi^2}{L^2}t}\right)+\sum_{n=1}^{\infty}\left(\cos\left[(2n-1)\frac{\pi}{L}x\right]e^{-\kappa(2n-1)^2\frac{\pi^2}{L^2}t}\right)\right)}{\left(\sum_{n=1}^{\infty}\left(\frac{1}{2n-1}\sin\left[(2n-1)\frac{\pi}{L}x\right]e^{-\kappa(2n-1)^2\frac{\pi^2}{L^2}t}\right)\right)^2}\right)\hat{\imath} \qquad \text{(S11)}$$

Here, $e^{-(x-L/2)/L}$ ($e^{-(L/2-x)/L}$) for $L/2 \le x \le L$ ($0 \le x \le L/2$) and the plus (minus) sign in the second term on the second member is for $L/2 \le x \le L$ ($0 \le x \le L/2$). The trend of $\mathbf{F}_k^{(2)}$ is very similar for the different chemical species with a strong variation throughout the cell especially for small time.

In our 1D model, also the diffusion of molecules inside the cell occurs symmetrically along the x direction (x and $-x$ flow), namely $\mathbf{J}_{D\,k} = (J_{D\,k}, 0, 0)$ where $J_{D\,k} = \frac{dX_{D\,k}}{dt}$ with $dX_{D\,k}$ the diffusion flow of molecules. $J_{D\,k}$ is the mass diffusion flow for the kth chemical species per unit time crossing a surface of area $A = L^2$ perpendicular to the diffusion direction. It is proportional to the spatial derivative of n_m (x,t), the number of moles of chemical species per unit volume (moles/m^3) where n_m (x,t) is generically expressed as a concentration, namely as $n_m(x,t) = N_m(x,t)/V$ with N_m the number of moles and V the volume of the solution. The diffusion equation written in the 1D case and under the assumption of uniform diffusion takes the form:

$$\frac{\partial n_{m\,k}(x,t)}{\partial t} = D_k \frac{\partial^2 n_{m\,k}(x,t)}{\partial x^2} \qquad \text{(S12)}$$

Here, D_k is the diffusion coefficient (m^2/s) of the kth chemical species. Under the initial and boundary conditions applied to chemical species

$$\begin{cases} n_{m\,k}(x,0) = \delta(x) & \text{initial condition} \\ n_{m\,k}(x \to \infty, t) = 0 \text{ for } t > 0 & \text{boundary condition} \end{cases} \qquad \text{(S13)}$$

we get the most simple solution to Equation (S12) in the form of a Gaussian distribution function

$$n_{m\,k}\left(x,t\right)=\frac{N_{m\,k}}{A}\frac{1}{\sqrt{4\pi D_k\,t}}e^{-\frac{\left(x-\frac{L}{2}\right)^2}{4D_k\,t}} \tag{S14}$$

According to the initial condition in Equation (S13), it is reasonably assumed that a pulse of solute at t = 0 is present at a given point x that in our special case corresponds to the center of the cell x = L/2. In the special case, the solutes are the molecules that take part either in the cell respiration or in the lactic acid fermentation process. The boundary condition $n\left(x\to\infty,t\right)=0$ is realistically satisfied for a value of x close to the cell membrane.

The mass diffusion flow $J_{D\,k}$ per unit time of the kth molecule (number of moles $N_{m\,k}$ of the molecule per unit time crossing the surface of area A perpendicular to the flow) expressed in moles/(m^2 s) takes the form $J_{D\,k}(x,t)=-D_k\frac{\partial n_{m\,k}(x,t)}{\partial x}$. The minus sign only indicates that diffusion is from the region at higher concentration to that at lower concentration. Unlike the heat flow, the mass diffusion flow has a dependence on the chemical species considered. Explicitly, by performing the spatial derivative of Equation (S15), we get:

$$\mathbf{J}_{D\,k}\left(x,t\right)=\pm\frac{N_{m\,k}}{A}\frac{1}{4\sqrt{\pi D_k}}\frac{\left(x-L/2\right)}{t^{3/2}}e^{-\frac{\left(x-\frac{L}{2}\right)^2}{4D_k t}}\,\hat{i} \tag{S15}$$

Here, the plus (minus) sign is referred to the diffusion flow either along $+x$ ($L/2 \le x \le L$) or along $-x$ ($0 \le x \le L/2$) being the flow bidirectional.

In Fig. S4, we draw the diffusion flow expressed in equation (S15) for the reagents of the respiration process, viz. one mole of glucose ($N_{m\ C6H12O6}$ = 1) and six moles of oxygen ($N_{m\ O2}$ = 6) in both a normal and a cancer cell. We have carried out the calculations taking the diffusion coefficients from Tab.1 of the main text. For both chemical species the flow is of comparable magnitude, exhibits a peak close to the centre of the cell, with a broader distribution close to the centre in a normal cell especially in the first instants of time, and then a decrease going towards the border and with increasing time.

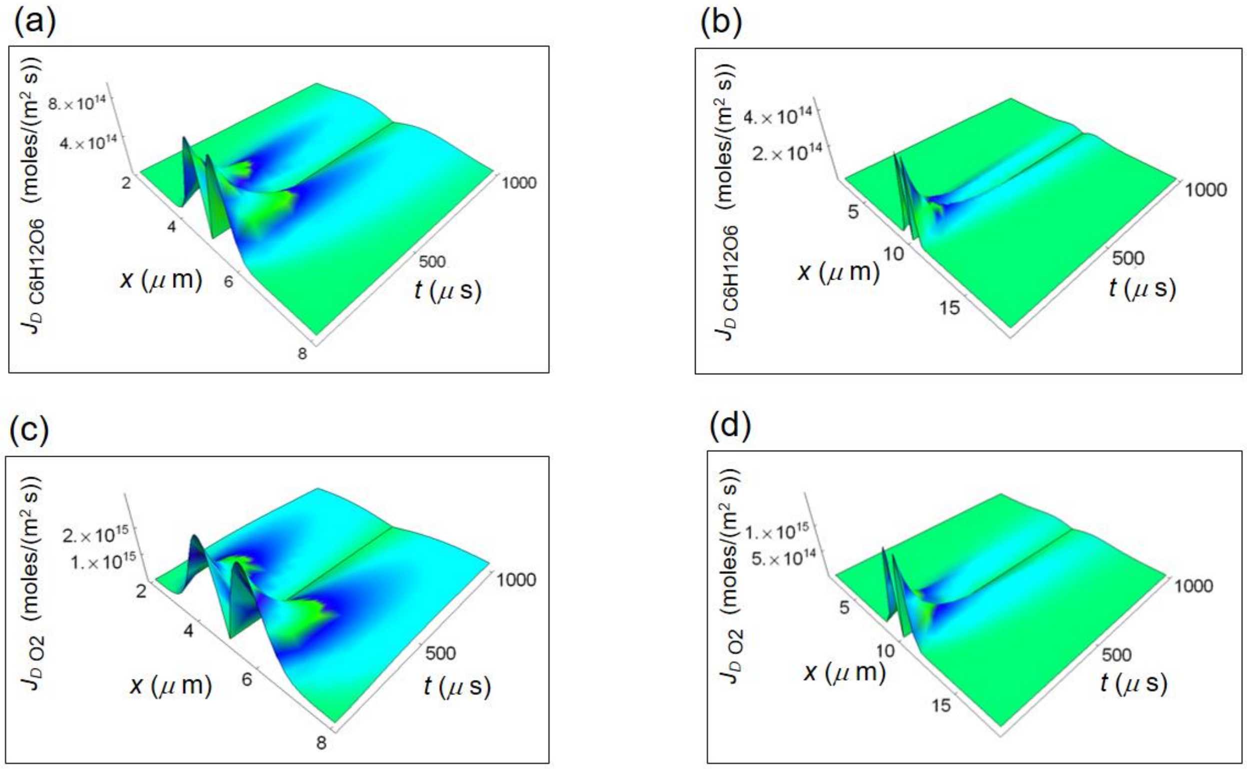

Figure S4. Diffusion flow of the reagents involved in the glucose catabolism according to equation (S15). (a) Diffusion flow of one mole of glucose molecule in a normal cell. (b) Diffusion flow of one mole of glucose molecule in a cancer cell. (c) Diffusion flow of six moles of oxygen molecule in a normal cell. (d) Diffusion flow of six moles of oxygen in a cancer cell.

In Fig. S5, we show the diffusion flow of the products of the respiration process in a normal and in a cancer cell. The behaviour is very similar to that of the reagents.

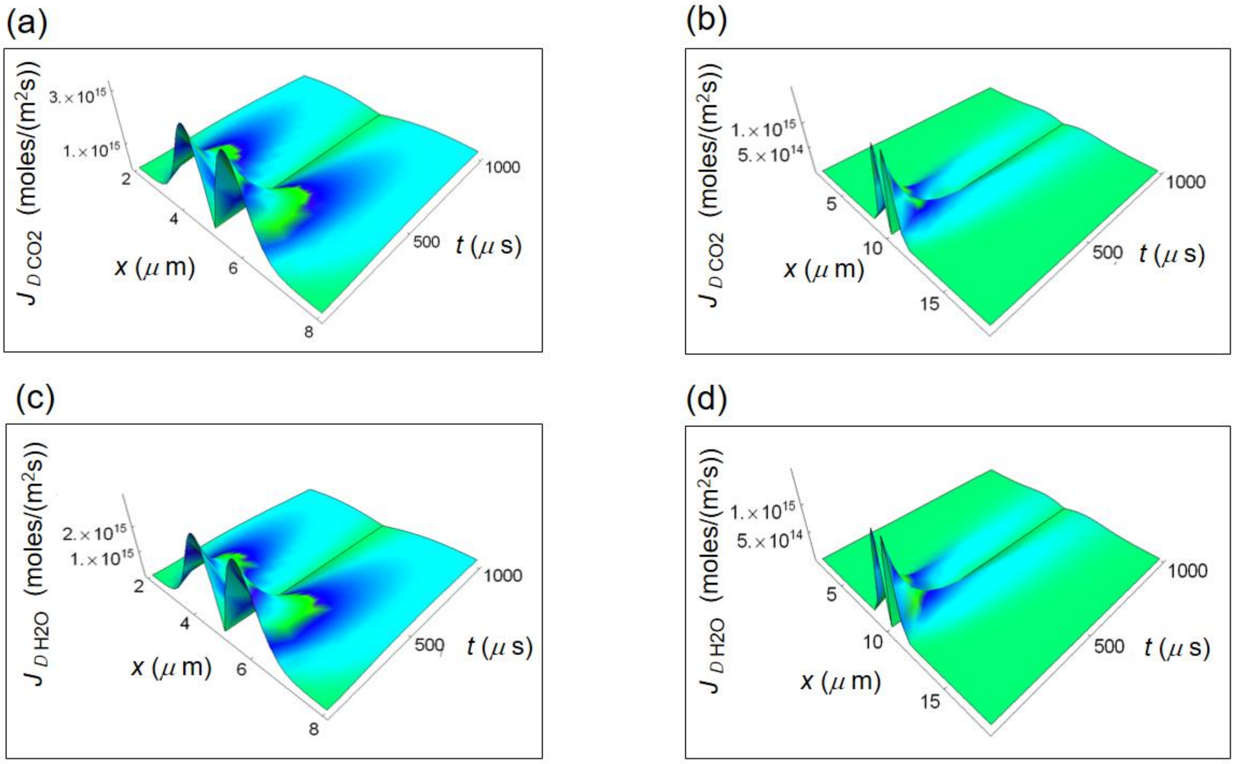

Figure S5. Diffusion flow of the products of the respiration in a cell according to equation (S15). (a) Diffusion flow of six moles of carbon dioxide molecule in a normal cell. (b) Diffusion flow of six moles of carbon dioxide molecule in a cancer cell. (c) Diffusion flow of six moles of water in a normal cell. (d) Diffusion flow of six moles of water in a cancer cell.

Fig. S6 displays the diffusion of the products of the lactic acid fermentation. Because of the large value of the diffusion constant, the diffusion flow of the hydrogen ions is significant throughout the cell during the first instants of times. Also for these chemical species, there is a decrease of the diffusion flow with increasing time.

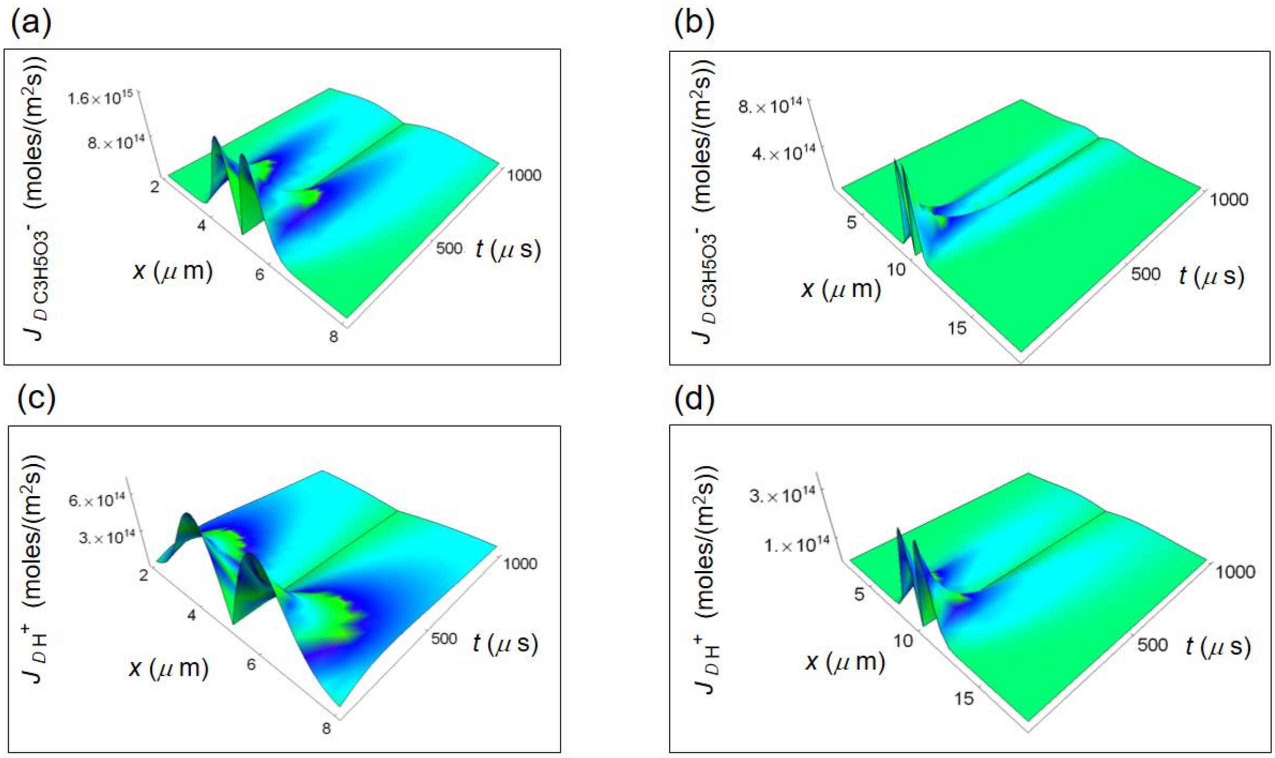

Figure S6. Diffusion flow of the products of the lactic fermentation process according to equation (S15). (a) Diffusion flow of two moles of lactate ions in a normal cell. (b) Diffusion flow of two moles of lactate ions in a cancer cell. (c) Diffusion flow of two moles of hydrogen ions in a normal cell. (d) Diffusion flow of two moles of hydrogen ions in a cancer cell.

By means of equations (S11) and (S15), we get the general expression of $r_{i\,D}\,(x,t)$ valid for any irreversible processes, viz.

$$r_{i\,D}(x,t) = \frac{\pi^{\frac{3}{2}}}{16}\frac{1}{T_0}\frac{1}{V_{\text{cell}}}\frac{(x-L/2)e^{-\frac{t}{\tau}}}{t^{3/2}}\times$$

$$\sum_{k=1}^{N}\left(u_k\frac{N_{m\,k}}{\sqrt{D_k}}e^{-\frac{(x-L/2)^2}{4D_k t}}\right)\left(\frac{\left(\sum_{n=1}^{\infty}\left(\cos\left[(2n-1)\frac{\pi}{L}x\right]e^{-\kappa(2n-1)^2\frac{\pi^2}{L^2}t}\right)e^{-|x-L/2|/L}\right)}{\left(\sum_{n=1}^{\infty}\left(\frac{1}{2n-1}\sin\left[(2n-1)\frac{\pi}{L}x\right]e^{-\kappa(2n-1)^2\frac{\pi^2}{L^2}t}\right)\right)^2}\right) \quad \text{(S16)}$$

after having neglected the term proportional to the sine series at the numerator that is much smaller than the one proportional to the cosine series. Equation (S16) is equation (2) of the main text.

In particular, equation (S16) applied to glucose catabolism in normal and cancer cells reads

$$r_{i\,D}(x,t) = \frac{\pi^{\frac{3}{2}}}{16}\frac{1}{T_0}\frac{1}{V_{\text{cell}}}\frac{(x-L/2)e^{-\frac{t}{\tau}}}{t^{3/2}}\times$$

$$\left(w_{\text{resp}}\sum_{k=1}^{N_{\text{resp}}}\left(u_k\frac{N_{m\,k}}{\sqrt{D_k}}e^{-\frac{(x-L/2)^2}{4D_k t}}\right)+w_{\text{ferm}}\sum_{k=1}^{N_{\text{ferm}}}\left(u_k\frac{N_{m\,k}}{\sqrt{D_k}}e^{-\frac{(x-L/2)^2}{4D_k t}}\right)\right)\left(\frac{\left(\sum_{n=1}^{\infty}\left(\cos\left[(2n-1)\frac{\pi}{L}x\right]e^{-\kappa(2n-1)^2\frac{\pi^2}{L^2}t}\right)e^{-|x-L/2|/L}\right)}{\left(\sum_{n=1}^{\infty}\left(\frac{1}{2n-1}\sin\left[(2n-1)\frac{\pi}{L}x\right]e^{-\kappa(2n-1)^2\frac{\pi^2}{L^2}t}\right)\right)^2}\right) \quad \text{(S17)}$$

with w_{resp} and w_{ferm} the probability weights associated to respiration and fermentation processes, respectively with $w_{\text{ferm}} = 1-w_{\text{resp}}$ and N_{resp} and N_{ferm} the corresponding numbers of chemical species and $r_{i\,D}(x,t)\geq 0$.

C. RATE OF ENTROPY DENSITY PRODUCTION DUE TO IRREVERSIBLE CHEMICAL REACTIONS

We now study the term contributing to the RIEDP due to the irreversible chemical reactions. From Equation (S1) this contribution takes the general form:

$$r_{i\,r}(\mathbf{x},t) = \frac{1}{T(\mathbf{x},t)}\sum_{j=1}^{M}A_j(\mathbf{x},t)\,\mathrm{v}_j \quad \text{(S18)}$$

Here, the subscript "r" stands for reactions, the affinity of the jth reaction reads $A_j(\mathbf{x},t) = -\sum_{k=1}^{N}\nu_{k\,j}\mu_k(\mathbf{x},t)$ with $\nu_{k\,j}$ the stoichiometric coefficients, N is the number of chemical

species, $\mu_k(\mathbf{x},t)$ is the space and time dependent chemical potential and $\mathrm{v}_j = \frac{1}{V_{\mathrm{cell}}}\frac{d\xi_j}{dt}$ is the velocity of the jth reaction, viz. the derivative of the jth degree of advancement $d\xi_j$ with respect to time divided by V_{cell}. In this framework, the affinity plays the role of the thermodynamic force and the velocity that of the corresponding thermodynamic flow associated to irreversible reactions.

In our analysis, we set $M = 1$ for every chemical reaction and we assume that flows of molecules are along the x direction so that

$$r_{i\,r}(x,t) = \frac{1}{T(x,t)} A(x,t)\,\mathrm{v}_1 \tag{S19}$$

The affinity of every irreversible reactions taking place in the cell cytoplasm takes the form

$$A(x,t) = -\sum_{k=1}^{N} v_k\, u_k\, e^{-\left(|x-L/2|/L+t/\tau\right)} \tag{S20}$$

and is expressed in J/moles. Instead, the corresponding velocity is $\mathrm{v} = \frac{1}{V_{\mathrm{cell}}}\frac{d\xi}{dt} > 0$ with $d\xi$ the variation of the degree of advance of the reaction and is in moles/ (m^3 s). Irreversible chemical reactions occurring in cells are either second-order or first order. The most general expression of the velocity (rate) for a second-order reaction of the form l A + m B $\rightarrow$ f C with l, m and f the number of moles of A, B and C, respectively is

$$\mathrm{v} = \mathrm{k}_{\mathrm{kin}}\, n_{\mathrm{A}}\, n_{\mathrm{B}} \tag{S21}$$

where, $k_{\mathrm{kin}} = K\, e^{-\frac{E_a}{RT_0}} > 0$ is the kinetic constant (subscript "kin" stands for kinetic) of the velocity of the reaction expressed in 1/(M s) where M is the molarity, E_a the activation energy and R = 8.314472 J/(mole K) the gas constant with K the pre-exponential factor and $n_{\mathrm{A}} = N_{m\,\mathrm{A}}/V$ ($n_{\mathrm{B}} = N_{m\,\mathrm{B}}/V$) is the molar concentration of reagents A (B) with $N_{m\,\mathrm{A}}$ ($N_{m\,\mathrm{B}}$) the number of moles of A (B). The velocity depends on the molar concentration of the two chemical species expressed by the reagent A and B. If B = 0 (A = 0) the velocity of a second-order reaction reduces to $\mathrm{v} = \mathrm{k}_{\mathrm{kin}} n_{\mathrm{A}}^2$ ($\mathrm{v} = \mathrm{k}_{\mathrm{kin}} n_{\mathrm{B}}^2$). Instead, in a first-order reaction velocity depends only on the molar concentration of a reagent

$$\mathrm{v} = \mathrm{k}_{\mathrm{kin}}\, n_{\mathrm{A}} \tag{S22}$$

with $\mathrm{k}_{\mathrm{kin}}$ expressed in 1/s.

Combining equations (S19), (S20) and the expression of the velocity of a reaction (equation (S21) and (S22)), we write down the RIEDP due to irreversible chemical reactions

$$r_{i\ r}(x,t) = -\frac{\pi}{4}\frac{1}{T_0}\frac{1}{V_{\text{cell}}^{p+q}}\frac{k_{\text{kin}}\sum_{k=1}^{N}\nu_k u_k e^{-\left(\left|x-L/2\right|/L+t/\tau\right)} N_{m\text{A reag}}^{\,p} N_{m\text{B reag}}^{\,q}}{\sum_{n=1}^{\infty}\left(\frac{\sin\left[(2n-1)\frac{\pi}{L}\left(\frac{L}{2}-x\right)\right]}{2n-1}e^{-\kappa(2n-1)^2\frac{\pi^2}{L^2}t}\right)} \tag{S23}$$

Here p=0, 1, 2, q = 0, 1, 2 and $p + q$ = 1, 2 for first- and second-order irreversible chemical reactions, respectively and $N_{m\ \text{A reag}}/V_{\text{cell}}$ ($N_{m\ \text{B reag}}/V_{\text{cell}}$) is the molar concentration of reagents A and B, respectively taking the volume of the solution equal to V_{cell}.

We now apply this formalism to glucose catabolism given by a sequence of reactions of glucose catabolism, either as respiration or as lactic acid fermentation process, classified as first-order. For this special case, we write $\text{v} = \frac{1}{V_{\text{cell}}}\frac{d\,N_{m\ C_6H_{12}O_6}/\nu_{c_{C_6H_{12}O_6}}}{dt} = -\frac{dn_{C_6H_{12}O_6}}{dt} > 0$ being $d\,N_{m\ C_6H_{12}O_6}/\nu_{c_{C_6H_{12}O_6}} = d\xi$ with $\nu_{c_{C_6H_{12}O_6}} = -1$ the glucose stoichiometric coefficient and $d\,n_{C_6H_{12}O_6} = \frac{1}{V_{\text{cell}}}dN_{m\ C_6H_{12}O_6} < 0$ the variation of glucose molarity (variation of the concentration of glucose moles in the solution of volume equal to V_{cell}) with $N_{m\ C_6H_{12}O_6}$ the number of moles of glucose (alternatively, one could choose $-\frac{1}{6}\frac{dn_{O_2}}{dt}$ with n_{O_2} the molar concentration of the oxygen, the second reagent of the glycolytic process). In the special case, $N_{m\,C_6H_{12}O_6} = 1$ and $dN_{m\,C_6H_{12}O_6} = -1$ in all studied reactions.

Specifically, $N_{m\ C_6H_{12}O_6} = N^{C_6H_{12}O_6}/N_A$ where $N^{C_6H_{12}O_6}$ is number of glucose molecules and N_A = 6.02 $\times 10^{23}$ is the Avogadro number. Explicitly, the affinity of glucose catabolism reads

$$A(x,t) = -\left(w_{\text{resp}}\sum_{k=1}^{N_{\text{resp}}}\nu_k u_k e^{-\left(\left|x-L/2\right|/L+t/\tau\right)} + w_{\text{ferm}}\sum_{k=1}^{N_{\text{ferm}}}\nu_k u_k e^{-\left(\left|x-L/2\right|/L+t/\tau\right)}\right) \tag{S24}$$

Here, w_{resp} and w_{ferm} are the probability weights associated to respiration and fermentation processes, respectively with w_{ferm} = 1–w_{resp} and N_{resp} and N_{ferm} are the corresponding numbers of chemical species.

In Fig. S7, we plot the affinity for a normal cell (panel (a)) and for a cancer cell (panel (b)). In the numerical calculations, we have taken the following parameters: w_{resp} = 0.8 (0.1) and w_{ferm} = 0.2 (0.9) for a normal (cancer) cell [5], $\tau = 10^{-4}$ s, $k_{kin} = 10^{-4}$/s for normal cells and $k_{kin} = 10^{-5}$/s for cancer cells [S6]. The values used for the chemical potentials at $x = L/2$ and $t = 0$ (partial molar energy) of the different chemical species are the ones in Table 1 of the main text.

In both cases, the affinity tends to zero with increasing time as should be expected for every thermodynamic system moving towards equilibrium. Looking at Fig. S7, it turns out that the affinity for a normal cell is one order of magnitude greater than the corresponding one for a cancer cell. This means that the thermodynamic force associated to the glucose catabolism reaction in a cancer cell is weaker with respect to that of the corresponding normal cell.

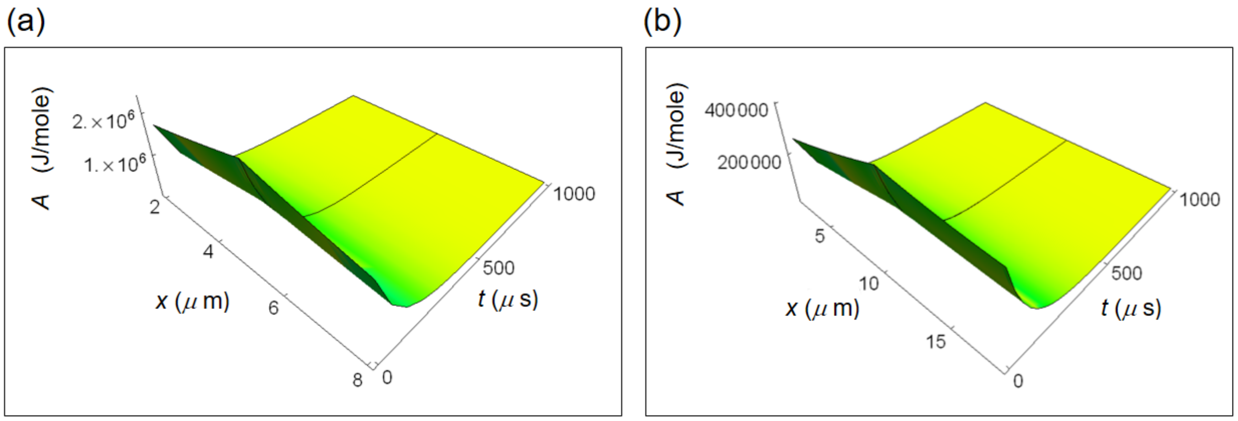

Figure S7. Affinity for a cell calculated by means of equation (S24). (a) Affinity for a normal cell. (b) Affinity for a cancer cell.

Like for other first-order reactions, we write the velocity of the glucose catabolism in the form

$$\mathrm{v} = k_{\mathrm{kin}}^{\mathrm{resp\ (ferm)}} n_{C_6H_{12}O_6} \tag{S25}$$

with $k_{\mathrm{kin}}^{\mathrm{resp\ (ferm)}} > 0$ the pathway "kinetic constant" of the respiration (fermentation) process expressed in 1/s.

For the case of glucose catabolism that can be regarded as a first-order reaction, equation (S23) becomes

$$r_{i\ r}(x,t) = -\frac{\pi}{4}\frac{1}{T_0}\frac{1}{V_{\text{cell}}}\times \frac{k_{\text{kin}}\left(w_{\text{resp}}\sum_{k=1}^{N_{\text{resp}}}\nu_k u_k e^{-\left(\left|x-L/2\right|/L+t/\tau\right)} N_{m\ C_6H_{12}O_6} + w_{\text{ferm}}\sum_{k=1}^{N_{\text{ferm}}}\nu_k u_k e^{-\left(\left|x-L/2\right|/L+t/\tau\right)} N_{m\ C_6H_{12}O_6}\right)}{\sum_{n=1}^{\infty}\left(\frac{\sin\left[(2n-1)\frac{\pi}{L}\left(\frac{L}{2}-x\right)\right]}{2n-1}e^{-\kappa(2n-1)^2\frac{\pi^2}{L^2}t}\right)} \tag{S26}$$

by taking into account the mixed nature of glucose catabolism occurring in both types of cells (both respiration and fermentation process). Here, $e^{-\frac{(L/2-x)}{L}}$ for $0 \le x \le L/2$ and $e^{-\frac{(x-L/2)}{L}}$ for $L/2 \le x \le L$ and $r_{i\ r}(x,t) \ge 0$. The subscript r stands for "irreversible reactions". Equation (S26) is equation (3) of the main text.

For both normal and cancer cell, it is always $\lim_{t\to\infty} r_i(x,t) = 0$, namely each contribution to the RIEDP vanishes when the thermodynamic system reaches the global equilibrium.

3. RATE OF EXTERNAL ENTROPY DENSITY PRODUCTION

The human cell (either normal or cancer cell) behaves like an open thermodynamic system. This means that it exchanges energy and matter with the intercellular environment. In this respect, it is useful to define the rate $r_e(\mathbf{x},t)$ of external entropy density production (REEDP) giving the amount of local entropy density outside a cell in the intercellular environment. Specifically, the REEDP has a contribution related to heat diffusion linked to energy exchange between the cell and the intercellular environment and a contribution due to matter exchange with the intercellular environment in terms of variation of the number of moles. No terms related to diffusion flow are present. Like for the calculation of the RIEDP due to heat flow, for the calculation of the REEDP we reasonably suppose that the temperature of the intercellular environment is spatially non-uniform and has a time dependence seeking for a solution to the heat equation free from boundary conditions.

Strictly speaking, for a thermodynamic system the infinitesimal entropy density exchanged between the cell and the environment due to heat release is $ds_{e\,Q} = \frac{1}{V_{\text{cell}}}\frac{dQ}{T_{\text{ic}}(x,t)}$, where dQ = dU + p dV is the infinitesimal heat transfer with U the internal energy, p the pressure and $T_{\text{ic}}(x,t)$ the space and time dependent intercellular temperature distribution where the subscript "ic" stands for intercellular. Therefore, the external contribution to the infinitesimal entropy density consists of two terms, viz. $ds_{\text{e}} = ds_{eQ} + ds_{\text{e}\,r}$ with $ds_{\text{e}\,r}$ the mass contribution associated to irreversible exchanges of molecules with the intercellular environment.

In our 1D model, we express the REEDP as $r_e(x,t) = r_{e\,Q}(x,t) + r_{e\,\text{exch}}(x,t)$ where $r_{e\,Q}(x,t)$ is the REEDP associated to heat flow, while $r_{e\,\text{exch}}(x,t)$ is due to exchanges of matter of the cell with the intercellular environment where the subscript "exch" stands for exchange. This scheme is valid for both a normal and a cancer cell.

Explicitly

$$r_e(x,t) = \frac{1}{T_{\text{ic}}(x,t)}\left(\frac{du(x,t)}{dt} + \frac{1}{V_{\text{cell}}}p(x,t)\frac{dV}{dt}\right) - \frac{1}{T_{\text{ic}}(x,t)}\sum_{k=1}^{N_{\text{pr}}}\mu_k(x,t)\frac{d_e N_{m\,k}}{dt} \tag{S27}$$

Here, $u = U/V_{\text{cell}}$ is the internal energy density. The second term on the second member expresses the matter flow with the external environment being $d_e N_{m\,k}$ the variation of the number of moles of the products of the catabolic reaction and N_{pr} is the number of chemical species of products. By neglecting the small cell volume variation occurring during the entropy transfer ($dV/dt \approx 0$) we get

$$r_e(x,t) \simeq \frac{1}{T_{\text{ic}}(x,t)}\left(\frac{du(x,t)}{dt} - \sum_{k=1}^{N_{\text{pr}}}\mu_k(x,t)\frac{d_e N_{m\,k}}{dt}\right) \tag{S28}$$

A. RATE OF EXTERNAL ENTROPY DENSITY PRODUCTION ASSOCIATED TO HEAT FLOW

In an open thermodynamic system like the human cell (either normal or cancer), part of the entropy exchanged with the extracellular environment is proportional to the variation of produced heat

according to the entropy definition in a thermodynamic system and cannot be calculated according to the internal heat flow valid for determining the RIEDP.

Keeping in mind that water is the main component of a human cell, from a thermodynamic point of view a cell (either normal or cancer cell) can be described as a fluid. Moreover, irreversible chemical reactions occur in a vapor phase where vapor is a substance that is a mixture between a gaseous and a liquid phase at room temperature. Hence, without loss of generality, we describe heat exchange with the environment in a way similar to heat exchange for an ideal gas. Owing to this thermodynamic analogy, the cell energy is thus equivalent to the gas kinetic energy. Hence, in the thermodynamic limit, namely ideally assuming that the volume increases with the number of molecules that in a cell is very high, the cell energy has a continuous spectrum of values. Its derivation, in the simplest picture, starts from the partition function of a monoatomic gas. Note that water in the gaseous phase behaves not like a monoatomic gas but like a triatomic gas. However, without loss of generality, the kinetic energy due to the rotation and vibration of water molecules typical of polyatomic molecules is much smaller than the translational kinetic energy that is instead associated to heat flow and this justifies the analogy with a monoatomic gas where only translational degrees of freedom are present. Moreover, within this description we do not take into account also the potential energy due to intermolecular forces among water molecules that does not play a role in heat flow. Straightforwardly, the partition function Z of an ideal monoatomic gas, that is equivalent within this description to the cell partition function, is expressed by the well-known formula $Z = A\,T^{\frac{3}{2}}$ where $A = V_{\text{cell}}\,(2\,k_{\text{B}}\,m)^{3/2}/h^3$ with $k_{\text{B}} = 1.3805 \times 10^{-23}$ J/K the Boltzmann constant, m the mass and h the Planck constant [S7]. From the well-known relation between the average energy and the partition function, viz. $\bar{E} = k_{\text{B}} T^2 \dfrac{d}{dT} \ln Z$ we get $U(x, t) = 3/2\, N\, k_{\text{B}}\, T(x, t)$, where the internal energy is a thermal energy and has a space and time dependence getting its largest contribution from kinetic energy. Here, $U = N\,\bar{E}$ being N the number of molecules carrying the kinetic energy (translational and vibrational) and $N = N_m\, N_{\text{A}}$ where N_m is the number of moles of the products of glucose catabolism (respiration process for the normal cell and lactic fermentation for the cancer cell) and $N_{\text{A}} = 6.02 \times 10^{23}$ molecules is the Avogadro number. As expected, the internal energy of the cell due to heat exchange with the intercellular environment turns out to be proportional to T and depends on time t via the temperature.

We write down the well-known fundamental solution to the heat equation (equation (S3)) obtained with no boundary conditions since we are considering the intercellular environment and we are

making some consideration about the finite heat quantity flowing towards the intercellular environment, viz.

$$T_{\mathrm{ic}}\left(x,t\right)=\frac{T_{\mathrm{ic}}\,x_0}{\sqrt{4\pi\kappa t}}e^{-\frac{\left(x-L\right)^2}{4\kappa t}} \tag{S29}$$

Here, $x > L$, T_{ic} is the maximum intercellular temperature and x_0 is a characteristic length in the intercellular environment over which the temperature varies. In the model calculations, we have taken the realistic value x_0 = 10 nm such that the temperature in the intercellular space at the border with the cell and especially at the initial instant of times when the release of heat takes place is about 310 K. We show the temperature distribution in the intercellular environment of both a normal and a cancer cell in Fig. S8.

Although in some cancer tissues the separation between adjacent cells vanishes because of the tumor, we have taken the value of the separation of about 0.2-0.3 μm between two adjacent normal cells and about 1.5 μm between two cancer cells considering the typical values for the epithelial cells of human breast tissue [S8]. There is a maximum temperature close to the border between the cell and the intercellular environment and T_{ic} tends to decrease slightly by going away from the cell. Of course, for a realistic description we should take into account also the temperature distribution of the adjacent cell (not shown) but it would be outside the aims of this approach that focuses on the thermodynamic behaviour of a single cell.

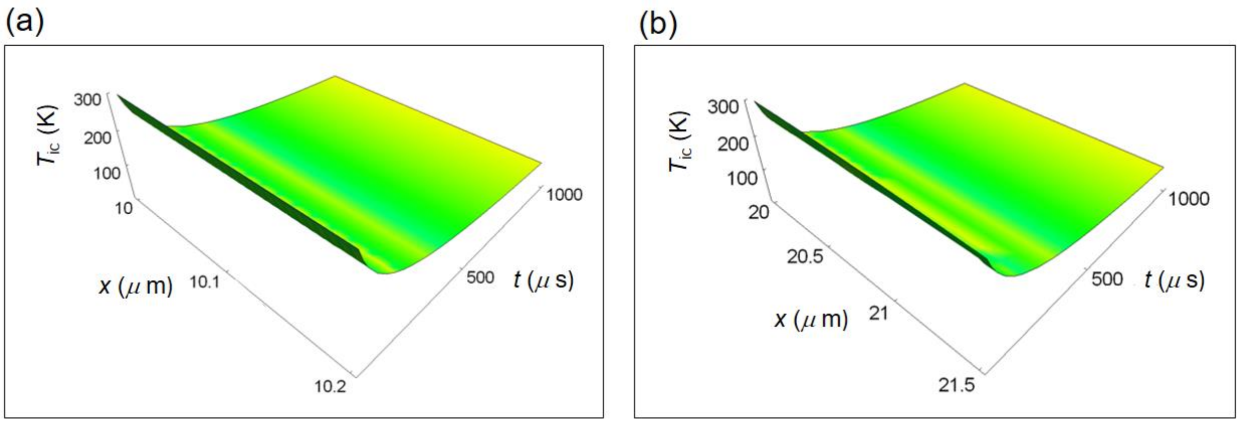

Figure S8. Intercellular temperature distribution calculated according to equation (S3) and expressed in equation (S29). (a) Temperature distribution in the intercellular environment for a normal cell. (b) Temperature distribution in the intercellular environment for a cancer cell.

The time derivative, calculated via the expression $U(x, t) = 3/2\ N\ k_B\ T_{ic}(x,t)$, reads approximately $\frac{d\,u(x,t)}{dt} \approx \frac{1}{V_{cell}} \frac{3}{2} k_B N \frac{T_{ic}\ (x,t)\left(\kappa(x-L)^2\right)\sqrt{4\pi\kappa t}}{8(\kappa t)^2\sqrt{\pi\kappa t}}$ taking into account that, for x large and small t, $\kappa(x-L)^2 >> \kappa^2 t$. The REEDP related to heat transport from the cell to the intercellular environment is approximately

$$r_{eQ}(x,t) \approx \frac{1}{T_{ic}(x,t)} \frac{du(x,t)}{dt} \tag{S30}$$

We substitute $\frac{du(x,t)}{dt}$ getting

$$r_{eQ}(x,t) \simeq \frac{1}{V_{cell}} \frac{3}{8} k_B \frac{N_A N_{m\,pr}}{\kappa} \frac{(x-L)^2}{t^2} \tag{S31}$$

where, $N_{m\,pr}$ are the number of moles of the products for the studied irreversible chemical reaction.

Equation (S31) is general and valid for describing r_{eQ} associated to external heat transport due to irreversible processes in normal and cancer cells. Equation (S31) is equation (4) of the main text. If applied to glucose catabolism we need to take into account the weights associated to respiration and fermentation processes yielding

$$r_{eQ}(x,t) \simeq \frac{1}{V_{cell}} \frac{3}{8} k_B \frac{N_A\left(N_{m\,pr\,resp} w_{resp} + N_{m\,pr\,ferm} w_{ferm}\right)}{\kappa} \frac{(x-L)^2}{t^2} \tag{S32}$$

$N_{m\,pr\,resp}\left(N_{m\,pr\,ferm}\right)$ is the number of moles of the products in respiration (fermentation) process.

B. IRREVERSIBLE EXCHANGES WITH THE INTERCELLULAR ENVIRONMENT

The REEDP due to mass transport related to irreversible exchanges with the intercellular environment for any irreversible reaction occurring either in a normal or in a cancer cell is

$$r_{e\ \mathrm{exch}}\left(x,t\right)=-\frac{1}{T_{\mathrm{ic}}\left(x,t\right)}\frac{1}{V_{\mathrm{cell}}}\sum_{k=1}^{N_{\mathrm{pr}}}\mu_k\left(x,t\right)\frac{d_eN_{m\,k}}{dt} \tag{S33}$$

where $d_eN_{m\,k}$ is the variation of the number of moles of the kth product of the irreversible reaction with the subscript "e" indicating external and N_{pr} is the number of products with "pr" labelling products.

Substituting the expressions of the intercellular temperature distribution $T_{\mathrm{ic}}\left(x,t\right)$ given in equation (S29), of the chemical potential $\mu_k\left(x,t\right)=u_k e^{-\left(|x-L/2|/L+t/\tau\right)}$ and inserting the time $d\tau_1$ that is a characteristic time of the order of the inverse of k_{kin}, we get

$$r_{e\ \mathrm{exch}}\left(x,t\right)=-\frac{1}{T_{\mathrm{i\,c}}}\frac{1}{V_{\mathrm{cell}}}\frac{\sqrt{4\pi\kappa}}{x_0}t^{\frac{1}{2}}\,e^{\frac{\left(x-L\right)^2}{4\kappa t}}\sum_{k=1}^{N_{\mathrm{pr}}}u_k\,e^{-\left(|x-L/2|/L\,+t/\tau\right)}\frac{d_eN_{m\,k}}{d\tau_1} \tag{S34}$$

Equation (S34) is equation (5) of the main text.

In particular, for glucose catabolism $r_{e\ \mathrm{exch}}$ takes the general form

$$r_{e\ \mathrm{exch}}\left(x,t\right)=-\frac{1}{T_{\mathrm{ic}}\left(x,t\right)}\frac{1}{V_{\mathrm{cell}}}\left(w_{\mathrm{resp}}\sum_{k=1}^{N_{\mathrm{pr\ resp}}}\mu_k\left(x,t\right)\frac{d_eN_{m\ k\ \mathrm{resp}}}{dt}+w_{\mathrm{ferm}}\sum_{k=1}^{N_{\mathrm{pr\ ferm}}}\mu_k\left(x,t\right)\frac{d_eN_{m\,k\ \mathrm{ferm}}}{dt}\right) \tag{S35}$$

Here, $N_{\mathrm{pr\ resp}}$ ($N_{\mathrm{pr\ ferm}}$) is the number of products of respiration (fermentation) and $d_eN_{m\,k\ \mathrm{resp}}$ ($d_eN_{m\,k\ \mathrm{ferm}}$) is the variation of the number of moles moles of the products in the respiration (fermentation) process.

Substituting the expressions of $T_{\mathrm{ic}}\left(x,t\right)$ expressed in equation (S29) and of the chemical potential we get

$$r_{e\ \mathrm{exch}}\left(x,t\right)=-\frac{1}{T_0}\frac{1}{V_{\mathrm{cell}}}\frac{\sqrt{4\pi\kappa t}}{x_0}e^{\frac{\left(x-L\right)^2}{4\kappa t}}\times$$
$$\left(w_{\mathrm{resp}}\sum_{k=1}^{N_{\mathrm{pr\ resp}}}u_k\,e^{-\left(|x-L/2|/L\,+t/\tau\right)}\frac{d_eN_{m\ k\ \mathrm{resp}}}{d\tau_1}+w_{\mathrm{ferm}}\sum_{k=1}^{N_{\mathrm{pr\ ferm}}}u_k\,e^{-\left(|x-L/2|/L\,+t/\tau\right)}\frac{d_eN_{m\,k\ \mathrm{ferm}}}{d\tau_2}\right) \tag{S36}$$

where, $d\tau_1$ ($d\tau_2$) is a characteristic time such that $1/\,d\tau_1$ ($1/\,d\tau_2$) is about 10^{-5}/s (10^{-4}/s), namely of the order of the pathway kinetic constant of the glucose catabolism reaction in both processes.

By analysing the behaviour of the three contributions to REEDP it is $\lim_{t\to\infty} r_e\,(x,t) = 0$. Hence, we have proved that the equilibrium state of an open thermodynamic system like a cell (either normal or cancer), where irreversible processes take place, implies the minimization of both r_i and r_e with increasing time and its vanishing in the limit of infinite time.

References

S1. Kondepudi, D. & Prigogine, I. *Modern thermodynamics: From heat engines to dissipative structures* (Wiley, 2015).

S2. Dolfi, C.S. *et al.* The metabolic demands of cancer cells are coupled to their size and protein synthesis rate. *Cancer & Metabol.* 1, 1-13 (2013).

S3. Apps, D.K. & Nairn, A.C. The equilibrium constant and the reversibility of the reaction catalysed by nicotinamide-adenine dinucleotide kinase from pigeon liver. *Biochem. J.* **167**, 87-93 (1977).

S4. Pessôa, C. A., Gushikem, Y. & Kubota, T. K. Ferrocenecarboxylic acid adsorbed on Nb_2O_5 film grafted on a SiO_2 surface: NADH oxidation study. *Electrochim. Acta* **46**, 2499-2505 (2001).

S5. Warburg, O. On respiratory impairment in cancer cells. *Science* **124**, 269–270 (1956).

S6. Beck, W.S. A kinetic analysis of the glycolytic rate and certain glycolytic enzymes in normal and leukemic leucocytes. *J. Biol. Chem.* **216**, 333-350 (1955).

S7. Alonso, M. & Finn, E.J. *Quantum and statistical physics Vol. 3* (Addison Wesley, 1968).

S8. Ozzello, L. Ultrastructure of human mammary carcinoma cells in vivo and in vitro. *J. Natl. Cancer Inst.* **48**, 1043-1050 (1972).
